# Unprotected and interconnected Ru^0^ nano-chain networks: advantages of unprotected surfaces in catalysis and electrocatalysis†
†Electronic supplementary information (ESI) available: Information on reagents, instrumentation and analytical techniques employed are elaborated. Detailed calculation of conversion, selectivity, yield, TON, TOF and the rate constants for catalytic nitroarenes hydrogenation is given. TOF calculation for the electrocatalytic water splitting is explained. Calibration curves for finding the real concentrations of the nitro compounds as Fig. S1A–F and the corresponding concentration are provided as Table S1. Details on the construction of calibration curves are given. XRD and EDS spectra are given as Fig. S2 and S3A–C. The detailed concentration of nitroarenes taken for catalytic study and other reaction parameters are tabulated as Table S2. The comparative interpretation for the catalytic activity of unprotected Ru^0^ nano-chain networks with the Ru catalysts in other forms and noble metal catalysts are provided as Tables S3 and S4. The LDPS particle size distribution is provided as Fig. S4A–C. The time dependent UV-Vis spectra and the corresponding first order kinetic plots for all nitroarenes except 4-NS are provided as Fig. S5A–M and N–Z. UV-Vis spectra, TEM micrographs and electron diffraction patterns of Ru-CTAB, Ru-SDS and Ru-TX-100 are provided as Fig. S6A–I. The time-dependent UV-Vis spectra for the hydrogenation of 4-NS by Ru-CTAB, Ru-SDS and Ru-TX-100 and their corresponding ln(conc.) *vs.* time plots are given as Fig. S7A–F. CV of Ru-30, Ru-45 and Ru-60 are given together as Fig. S8. Post-cycle CV for Ru-60 after 10 h of chronoamperometric analysis is given as Fig. S9. CV of Ru-60 modified GC with the potential window of –1 to 1 V for identifying the non-faradaic region is given as Fig. S10. See DOI: 10.1039/c5sc04714e


**DOI:** 10.1039/c5sc04714e

**Published:** 2016-01-20

**Authors:** S. Anantharaj, M. Jayachandran, Subrata Kundu

**Affiliations:** a Electrochemical Materials Science (ECMS) Division , CSIR-Central Electrochemical Research Institute (CECRI) , Karaikudi-630006 , Tamilnadu , India . Email: skundu@cecri.res.in ; Email: subrata_kundu2004@yahoo.co.in ; Fax: +91-4565-227651 ; Tel: +91-4565-241487

## Abstract

Seedless, surfactantless and support-free unprotected, metallic, interconnected nano-chain networks of ruthenium nanoparticles (NPs) were successfully synthesized *via* the reduction of ruthenium(iii) chloride (RuCl_3_) with sodium borohydride (NaBH_4_) at three different temperatures, *viz.* 30 °C, 45 °C and 60 °C. The molar ratio of RuCl_3_ solution and borohydride was optimized to be 1 : 1.5 to produce stable colloids with the optimum final solution pH of 9.7 ± 0.2. Average diameters of the interconnected nano-chain networks prepared at 30 °C (Ru-30), 45 °C (Ru-45) and 60 °C (Ru-60) were 3.5 ± 0.5 nm, 3.0 ± 0.2 nm and 2.6 ± 0.2 nm respectively. The morphology and composition dependent catalytic and electrocatalytic activities of these unprotected Ru nano-chain networks (Ru-30, Ru-45 and Ru-60) were studied in detail. The catalysis study was performed by investigating the transfer hydrogenation of several substituted aromatic nitro compounds. It was observed that Ru-60 was relatively more active compared to Ru-30 and Ru-45, which was reflected in their rate constant values. The electrocatalytic activities of Ru-30, Ru-45 and Ru-60 were screened for anodic water splitting in alkaline medium (0.1 M NaOH) and it was found that all of them showed almost the same activity which required an over-voltage of 308 ± 2 mV to obtain an anodic current density of 10 mA cm^–2^. The catalytic and electrocatalytic performances of these unprotected Ru^0^ networks were compared with Ru^0^ nanomaterials prepared under similar conditions with three different surfactants, *viz.* CTAB, SDS and TX-100, which revealed that unprotected Ru^0^ networks are better catalysts than those stabilized with surfactants. The superior catalytic and electrocatalytic performance is due to the availability of unprotected Ru^0^ surfaces. The present route may provide a new possibility of synthesizing other surfactant-free, unprotected metal colloids for enhanced catalytic and electrocatalytic applications.

## Abstract 2

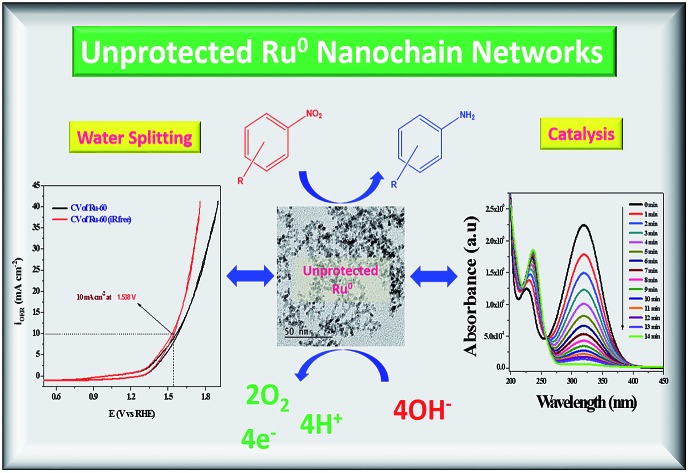
Surfactant- and support-free metallic, interconnected and unprotected Ru nano-chain networks are synthesized and screened for catalytic nitro arene hydrogenation and OER studies. Their excellent catalytic and electrocatalytic activities are due to the advantages of having unprotected Ru^0^ surfaces.

## Introduction

There have been many advances in the field of nanostructured zero-valent metals for the past two decades, and their interesting results, findings, methodologies and applications have made them a fascinating subject of research in the scientific community around the globe. Stabilizing metals at the nanoscale, and their subsequent applications in various areas such as electronics, optics, catalysis, energy conversion and in other emerging fields, has become an unavoidable and essential requirement in materials science. Among the various forms of metal based nanostructures, the syntheses of nanoparticles (NPs),^1^ thin films (TFs),^2^,^3^ nano-chains,^4^ nanowires (NWs),^5^ nanorods (NRs),^6^ and nanofoams (NFs)^7^ have been studied in depth by various physical and chemical routes. The chemistry of metal NPs such as Au,^8^ Ag,^5^ Pd^9^ and Pt^10^ with different morphologies has been studied in depth when compared to relatively more reactive metals such as Ir,^7^ Rh,^11^ Os,^12^,^13^ Cu,^14^ and Ru.^15^ Among the several transition metals with standard reduction potentials (*E*^0^) higher than that of hydrogen, Au, Ag, Pt and Pd are the most studied metals at the nanoscale level. Beyond the successful reduction and stabilization of metal NPs at the nano level, morphology, size and shape control are also essential for many specialized applications. To achieve control over the morphological parameters, many physical and chemical methods such as hydrothermal,^16^ sol–gel,^17^ laser ablation,^18^ physical vapor deposition (PVD),^19^ chemical vapor deposition (CVD),^20^ microwave assisted synthesis,^21^ sonochemical synthesis^22^,^23^ and wet chemical reductions^7^,^24^–^26^ have been employed. Among these methods , wet chemical reduction under various reaction conditions is the most preferred. As wet chemical synthesis is a bottom-up approach, it offers good control over the size and shape of metal nanostructures. The morphology selectivity or control is usually achieved by the use of additives such as stabilizers, surfactants, templates, scaffolds, layered materials and other 3D matrices. The nature of these additives varies from simple chemicals to bio-macromolecules such as DNA^27^ and cellulose,^28^ and even to naturally occurring 3D matrices like alumina, clays and minerals like ferrite and silica.^29^–^31^ All these materials basically offer selectivity over size, shape and morphology by acting as a structure determining platform for particle growth and as a host material. However, wet chemical routes to prepare metal nanomaterials with desired morphology without the addition of any such additives and external agents are highly limited, and have been reported only occasionally for the preparation of metal NPs such as Au,^32^–^34^ Pd,^35^ Pt,^36^ ternary metal nanocomposites like CuAgSe^37^ and some metal oxides.^38^,^39^ Obviously, it is highly desirable to prepare materials without any additives when it comes to practical applications, to avoid many unwanted losses in efficiency due to these additives. Moreover, a nanomaterial synthesized without any support and surfactant will offer a larger surface area compared to those with additives.

Among the transition metals, ruthenium has a positive standard electrode potential of 0.68 V (Ru^3+^ to Ru^0^ in water) that makes it possible to reduce at the nanoscale and stabilize for further applications. In bulk, it is a hard metal with a silvery white color. Moreover, it is a metal with a melting point higher than 2300 °C and a density of 12.41 g cm^–3^. Applications of Ru as the metal, metal oxide and complexes both in bulk and at the nanoscale level have been studied in catalysis and in electrocatalysis.^40^–^46^ There are many reports available for the synthesis of Ru metal nanomaterials with different morphologies for specific applications. Chau *et al.* reported β-cyclodextrin stabilized Ru NPs for hydrogenation reactions.^47^ Salas *et al.* reported the preparation of Ru NPs in ionic liquids.^48^,^49^ Stabilization of Ru NPs was achieved by heavily fluorinated compounds as reported by Tristany and co-workers.^50^ Ru NPs were prepared by a facile polyol reduction by Viau *et al.*^51^ In addition to these studies, reports on supported Ru metal NPs for catalytic applications are also available where β-zeolite,^52^ Fe_3_O_4_,^29^,^53^–^55^ graphene/reduced graphene oxide,^15^,^56^ silica,^57^ rutile,^58^ montmorillonite clay,^31^ nanostructured carbon^59^ and polystyrene^60^ are the common solid supports. Ru metal based nanoalloys and composites have also been prepared and used for many specific applications.^61^–^67^ Besides, spherical Ru NPs, Ru and Pt–Ru metal nanowires^62^,^63^ and nano-chains^68^ have also been reported where surfactants were employed to stabilize them. However, there is no report of the synthesis and stabilization of Ru metal nano-chain networks without any external stabilizer such as a surfactant, solid support, scaffold, template or structure-directing agents. The only metal which is frequently reported at nanoscale without any external stabilizing agents is Au,^32^–^34^ for which the phrase ‘surfactant-free synthesis’ and the term ‘unprotected’ are often employed.

On considering its unique catalysis applications, Ru is one among the most used transition metals for catalysis along with Pt, Pd, Ni, Ir, Au and Ag. Ruthenium, as Ru^0^, RuO_2_, mononuclear complexes, multinuclear complexes, bimetallic NPs such Pt–Ru, Ru–Ir and as composites with some other metal oxides, has been used extensively in many catalytic studies.^62^,^65^–^67^,^69^–^71^ Among them, the important chemical transformations are hydrogenation of alkenes,^49^ arenes,^47^ carbonyl compounds^72^ and nitroarenes,^40^,^59^,^73^ *N*-alkylation of sulfonamides, sulfinamides and amines,^29^ conversion of syngas to isoparaffins,^52^ conversion of nitriles^54^ and Heck and Suzuki type coupling reactions.^74^ On the other hand, Ru, RuO_2_ and its bimetallic and oxide composites have been studied extensively as electrocatalysts for various reactions such as oxygen evolution reaction (OER), hydrogen evolution reaction (HER), HCl oxidation and methanol oxidation reaction.^61^–^64^,^66^,^67^,^75^ Water splitting by anodic oxidation with Ru electrocatalysts is a well documented electrocatalytic application in which it competes with the state-of-the-art catalyst Ir.^76^ Nowadays, non-noble metal catalysts are also used as anodes in water electrolyzers, but in our case the main reason for choosing Ru for OER is to emphasize the advantages of unprotected Ru^0^ surfaces over the protected ones. While considering the versatile applicability of Ru and its compounds, surfactant-free and any support-free Ru nanomaterials are highly desired and required as the surface areas offered by surfactant-free nanomaterials are obviously higher than those of other nanomaterials surrounded by scaffolds, surfactants, templates and supports. To the best of our knowledge, this is the first ever report on the synthesis of unprotected Ru metal nano-chain networks, which is surfactant-free and synthesized without any solid support or external stabilizer, and the examination of their catalytic and electrocatalytic performances.

In this article, for the first time, we present the facile and fast synthesis of surfactant- and support-free unprotected metallic Ru interconnected nano-chain networks with three different average chain diameters by a simple wet chemical reduction of RuCl_3_·*x*H_2_O solution with sodium borohydride at three different temperatures, *viz.* 30 °C, 45 °C and 60 °C. The molar ratios of RuCl_3_·*x*H_2_O solution and sodium borohydride solution were optimized to obtain a stable colloidal solution of Ru^0^ nano-chain networks and the optimum final solution pH in all three networks was found to be 9.7 ± 0.2. The eventual average chain diameters of Ru-30, Ru-45 and Ru-60 networks were 3.5 ± 0.5 nm, 3.0 ± 0.2 nm and 2.6 ± 0.2 nm respectively. The synthesized materials were characterized using all essential spectroscopic and microscopic techniques to elucidate the morphology and chemical nature of the samples, and a detailed discussion is of the results is reported. The catalytic performances of Ru-30, Ru-45 and Ru-60 were investigated by taking several different nitro compounds, *viz.* nitrobenzene, 4-nitrophenol (4-NP), 4-nitroaniline (4-NP), 4-nitrostyrene (4-NS), 2-nitrophenol (2-NP), 2-nitroaniline (2-NA) and 2-bromo-6-nitrotoluene (2-B-6-NT). The electrocatalytic activities of Ru-30, Ru-45 and Ru-60 nanomaterials for anodic water splitting for OER in alkaline medium were also tested and discussed.

## Experimental section

### Synthesis of interconnected Ru^0^ nano-chain networks

**Scheme 1:**
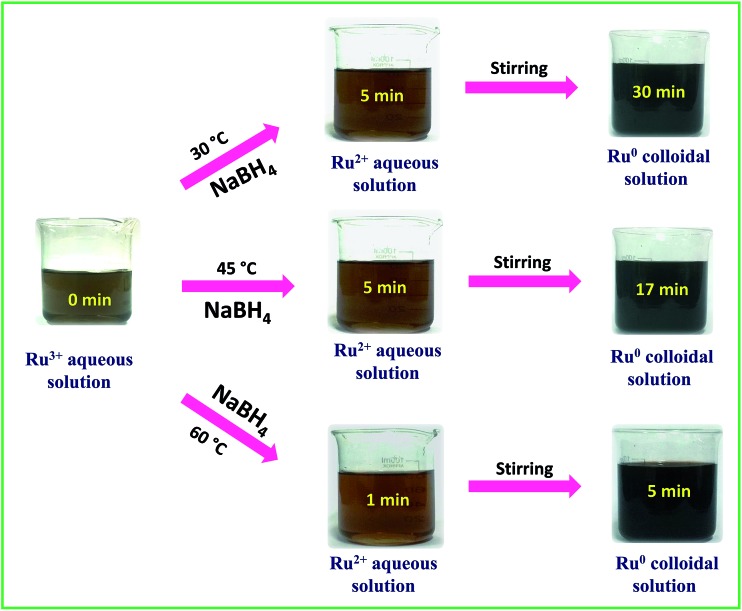
Time dependent snapshots taken during the synthesis of Ru-30, Ru-45 and Ru-60.

A stock solution of ruthenium(iii) chloride hydrate (RuCl_3_·*x*H_2_O) of concentration 0.01 M was prepared using Milli Q water. A solution of 0.1 M sodium borohydride was prepared freshly in ice-cold conditions and used for each trial. In a typical synthesis, the desired amount of Ru^3+^ stock solution was placed in a 250 mL beaker and stirred on a hot plate magnetic stirrer. Then the freshly prepared borohydride solution was added into the Ru^3+^ solution at a rate of 10 mL per 30 s. From the color change and the primary absorption spectral results the successful formation of Ru^0^ NPs was confirmed. To determine the optimum molar ratio between RuCl_3_·*x*H_2_O and borohydride to produce a stable colloidal solution of interconnected Ru^0^ nano-chain networks, the ratio was systematically changed and the results are shown in Table 1 along with the final pH of each solution. Depending on the reaction scheme, the rate of addition of borohydride was also varied wherever required. From Table 1, we can infer that at 30 °C, 45 °C and 60 °C, all Ru^3+^ : BH_4_^–^ molar ratios except for the molar ratio of 1 : 1.5 were found to be unable to produce a stable colloidal solution of interconnected Ru^0^ nano-chain networks. Depending on the molar ratio and the temperature, the aggregation and precipitation time of Ru^0^ particles varied from 1 min to 55 min. A stable dark-brown colloidal solution of interconnected Ru^0^ nano-chain networks was obtained at 30 °C, 45 °C and 60 °C when the ratio of metal precursor and borohydride was 1 : 1.5 and the final pH was about 9.7 ± 0.2. We also tried our synthesis at 75 °C and 90 °C, but as borohydride is a vigorously reacting reductant we experienced drastic spillage and bumping of the reaction mixture at both 75 °C and 90 °C. To ensure the complete reduction of Ru^3+^ to Ru^0^, the reaction mixtures were continuously stirred for 3 h further. To determine the stability of the colloidal solutions, a part from each solution was kept in the light under ambient conditions and another portion was kept in the dark and at 5 °C. The latter samples were found to be stable for more than a month and the ones kept in the light and in ambient conditions precipitated in 12–15 days. However, when the settled solutions were sonicated for 10 min, we obtained stable dark-brown colloidal solutions with stability that almost equalled that of the freshly prepared solutions. This implies that though the colloidal solutions are not stable for a long period of time, they can be made stable at any time by a simple sonication assisted re-dispersion. The catalytic performance was evaluated for the as-synthesized solutions of interconnected Ru^0^ nano-chain networks obtained at 30 °C, 45 °C and 60 °C. Electrocatalytic water splitting was carried out using interconnected Ru^0^ nano-chain network modified GC electrodes. The visible changes during the synthesis were captured and the complete synthesis sequence is depicted in Scheme 1.

**Table 1:** The detailed final concentrations and all other reaction parameters for the synthesis of Ru nano-chain networks

Trial No.	Ru^3+^ : BH_4_^–^ ratio	Final pH of the reaction mixture	Final conc. of Ru^3+^ (M)	Final conc. of BH_4_^–^ (M)	Temp. (°C)	Observation
1	2 : 1	5.81	5.0 × 10^–3^	2.5 × 10^–2^	30	Precipitated in 15 min
	2 : 1	5.93	5.0 × 10^–3^	2.5 × 10^–2^	45	Precipitated in 7 min
	2 : 1	5.91	5.0 × 10^–3^	2.5 × 10^–2^	60	Precipitated in 1 min
2	1 : 1	6.98	1.0 × 10^–3^	5.0 × 10^–2^	30	Precipitated in 25 min
	1 : 1	7.02	1.0 × 10^–3^	5.0 × 10^–2^	45	Precipitated in 37 min
	1 : 1	7.16	1.0 × 10^–3^	5.0 × 10^–2^	60	Precipitated in 55 min
3	1 : 1.5	9.88	**5.0 × 10^–4^**	**3.3 × 10^–2^**	**30**	**Stable colloid**
	1 : 1.5	9.73	**5.0 × 10^–4^**	**3.3 × 10^–2^**	**45**	**Stable colloid**
	1 : 1.5	9.92	**5.0 × 10^–4^**	**3.3 × 10^–2^**	**60**	**Stable colloid**
4	1.5 : 1	6.54	6.6 × 10^–3^	5.0 × 10^–3^	30	Precipitated in 20 min
	1.5 : 1	6.66	6.6 × 10^–3^	5.0 × 10^–3^	45	Precipitated in 28 min
	1.5 : 1	6.51	6.6 × 10^–3^	5.0 × 10^–3^	60	Precipitated in 35 min
5	1 : 2	11.23	2.5 × 10^–3^	5.0 × 10^–2^	30	Precipitated in 12 min
	1 : 2	11.35	2.55 × 10^–3^	5.0 × 10^–2^	45	Precipitated in 9 min
	1 : 2	11.41	2.55 × 10^–3^	5.0 × 10^–2^	60	Precipitated in 3 min
6	1 : 4	13.01	6.12 × 10^–4^	7.5 × 10^–2^	30	Precipitated in 7 min
	1 : 4	13.12	6.12 × 10^–4^	7.5 × 10^–2^	45	Precipitated in 3 min
	1 : 4	13.06	6.12 × 10^–4^	7.5 × 10^–2^	60	Precipitated in 1 min

### Catalytic hydrogenation of nitroarenes to aminoarenes

Hydrogenation of nitroarenes to aminoarenes is solely catalyzed by metals and metal NPs, and has been studied intensively with various catalysts and various nitro compounds. The catalytic performance of interconnected Ru^0^ nano-chain networks, *viz.* Ru-30, Ru-45 and Ru-60, were examined for the hydrogenation of seven different nitroarenes. In all these catalysis reactions, the volume of the catalyst solutions was kept about 20 μL. Similarly, the volume and concentration of the borohydride solution were also kept constant at about 100 μL and 0.1 M respectively. To find out the actual concentration of nitro compound at each point, calibration curves for all the nitro compounds were prepared using standard solutions of known concentration. The details of concentrations of each nitro compound are given in Table S1 (ESI†). The details of the volume and concentration of the nitro compounds are listed in Table S2 in ESI.† For a typical hydrogenation reaction of 4-nitrostyrene (4-NS) with these three catalysts, 0.5 mL of 10^–6^ M stock solution of 4-NS was placed in a glass vial of 15 mL volume into which 20 μL of Ru-30, Ru-45 or Ru-60 was added and the flask was shaken well for homogeneity. Then the volume of the reaction mixture was made up to 4 mL with water. Soon after adding a freshly prepared ice-cold solution of 100 μL of 0.1 M sodium borohydride, the mixture was shaken briefly and then about 3.5 mL of the solution was taken out into a quartz cuvette of path length 1 cm^2^ and analyzed using a double beam UV-Visible spectrophotometer. Time-dependent UV-Vis analyses at regular time intervals of 60 s were performed after the addition of borohydride. The resultant UV-Vis spectra of other nitroarene hydrogenation reactions were plotted together and are discussed in the Results and discussion section and in ESI.† The same procedure was followed for the hydrogenation of other nitroarenes with required changes in their concentration depending on their molar absorptivity.

### Electrocatalytic study of the interconnected Ru^0^ nano-chain networks by taking oxygen evolution as a test reaction

As well as the catalytic study of the hydrogenation of nitroarenes, the electrocatalytic water splitting ability of interconnected Ru^0^ nano-chain networks, *viz.* Ru-30, Ru-45 and Ru-60, was also examined. A GC electrode of 0.0732 cm^2^ area was taken as the bare working electrode. For better adhesion of our catalyst, binder Nafion (5%) solution was mixed with our catalyst solutions in the ratio of 1 : 9 by sonication. About 3 mg of Ru-30, Ru-45 or Ru-60 were homogenized with 1 mL of water and 5% Nafion (9 : 1) solution by sonication separately. About 5 μL of the resultant ink was cast carefully on the calibrated GC surface, ensuring a catalyst loading of 0.015 mg on each GC surface, and dried in ambient conditions for 10 h. In each modification, the catalyst loading was kept constant at about 0.205 mg cm^–2^. The modified GC electrodes were then used as working electrodes. About 20 mL of 0.1 M NaOH was taken with a Pt-foil counter electrode and an Hg/HgO reference electrode. The electrocatalytic activity was studied by running cyclic voltammetry (CV) at a scan rate of 10 mV s^–1^. Stability and kinetics were analyzed by chronoamperometry, steady state polarization techniques and electroimpedance spectroscopy and other required electrochemical characterization techniques. The results are discussed in detail in the Results and discussion section.

## Results and discussion

### UV-Visible spectroscopic study

**Fig. 1:**
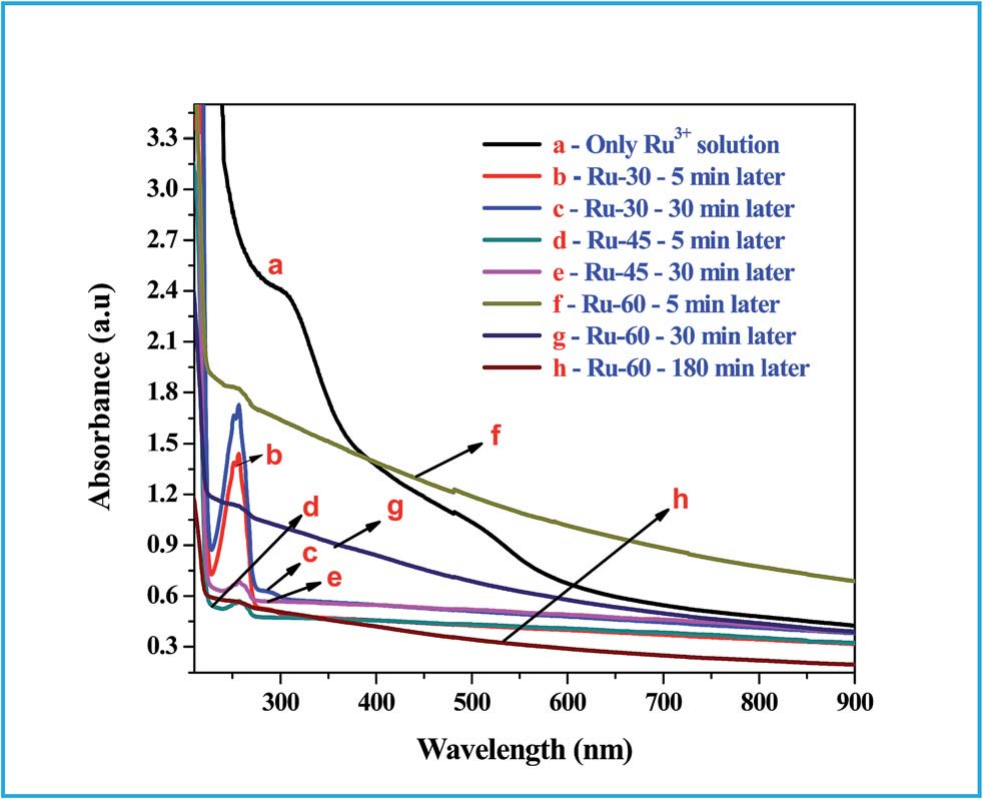
UV-Vis absorption spectra of Ru^3+^ solution and the reaction mixtures for the formation of interconnected unprotected Ru^0^ nano-chain networks.

UV-Visible absorption spectroscopic studies were performed to follow the chemical changes that occurred during and after the synthesis of colloidal Ru^0^ nanomaterials at different temperatures, *viz.* 30 °C, 45 °C and 60 °C. The absorption spectra for the metal ion precursor (Ru^3+^) and the synthesized colloidal Ru^0^ nanomaterials at different time intervals after the addition of borohydride were recorded and are given as Fig. 1. Curve a in Fig. 1 is the absorption spectrum of the Ru^3+^ solution in which two humps at 487 nm and 304 nm are observed which is as expected for Ru^3+^ solution and also in good agreement with earlier reports.^77^ Curves b, c are the absorption spectra of the reaction mixtures of Ru^3+^ solution and borohydride at 30 °C after 5 and 30 min from the addition of borohydride, respectively, and curves d, e are those at 45 °C. Curves b and d have a sharp and intense peak at 257 nm which is due to the formation of Ru^2+^ ion intermediates by the reduction of Ru^3+^ ions at the initial stage.^77^ Curves c and e are the absorption spectra of the same reaction mixtures after 30 min, where we can see a clear and sharp drop of almost 99% in the intensity of the Ru^2+^ peak observed at 257 nm. This clearly indicates that when the synthesis was performed at 30 °C and 45 °C, the initial reduction product was Ru^2+^ rather than Ru^0^, and on continued stirring further reduction of Ru^2+^ took place to form metallic Ru^0^ particles. However, in curves c and e a small hump is still observed, indicating unreacted Ru^2+^ ions. Curves f and g are the absorption spectra of the reactions performed at 60 °C: curve f was recorded 5 min after the addition of borohydride, and curve g was recorded at 30 min. Unlike the curves observed at 30 °C, the absorption spectra of the reaction mixture at 60 °C showed a gradual increase in the absorption while approaching the lower wavelength regions. This is attributed to interference caused by the rapid evolution of H_2_ gas from the reaction mixture as the temperature was high.^42^,^51^,^77^ This rapid evolution assisted the rapid reduction of Ru^3+^ to Ru^0^ within 5 min, unlike the reaction performed at 30 °C where it took more than 30 min. Even though the reduction was rapid and formed Ru^0^ in 5 min, a very small hump in curve f at 256 nm indicates the existence of some unreacted Ru^2+^ ions at this stage. When the stirring was further continued and temperature kept constant at about 60 °C for 30 min, the resultant absorption spectrum (curve g) had virtually no peak in the region characteristic of Ru^2+^ ions. Moreover, a total reduction in the absorption from the higher to the lower wavelength side can be observed, which is due to the reduced evolution of H_2_ gas from the reaction mixture. One more absorption spectrum of Ru-60 was taken after 3 h from the synthesis and given as curve h, which is of comparable intensity to those of Ru-30 and Ru-45. This in turn strongly supports our attribution to the increased intensity in the reaction mixture performed at 60 °C. The overall UV-Vis results showed the successful formation of metallic Ru^0^ particles by the reduction of Ru^3+^ solution by borohydride. These results are in good resonance with the earlier report of Li *et al.*^77^

### Transmission electron microscopic (TEM) studies

**Fig. 2:**
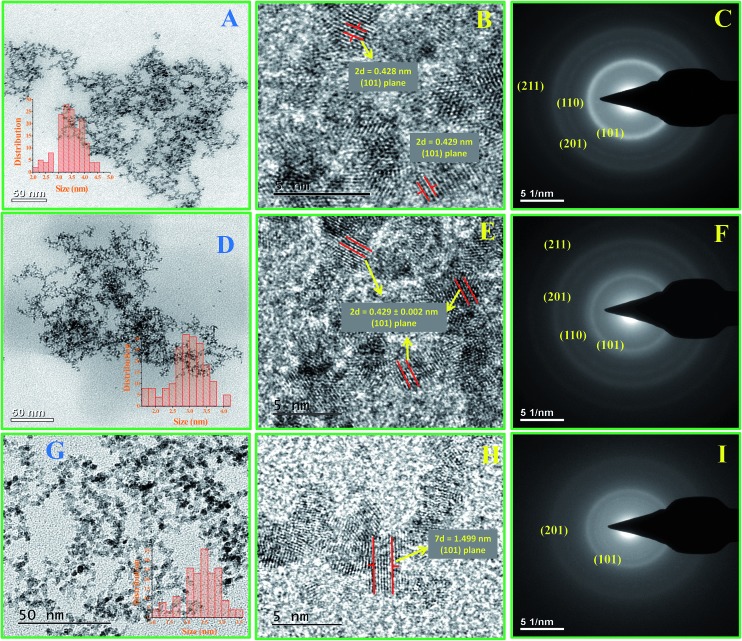
(A–I) TEM, HR-TEM micrographs and SAED patterns of the interconnected unprotected Ru^0^ nano-chain networks: (A–C) Ru-30, (D–F) Ru-45 and (G–I) Ru-60.

Transmission electron microscopic (TEM) and high resolution transmission electron microscopic (HR-TEM) studies were carried out to determine the morphology, size and nature of the materials and their fine structures. Fig. 2A–I shows the TEM, HR-TEM and SAED pattern images of Ru-30, Ru-45 and Ru-75. Fig. 2A, D and G reveal overall morphologies of Ru-30, Ru-45 and Ru-60 respectively at low magnification. It is clear from these images that all of them have similar morphologies of dense, interconnected networks of Ru^0^ nano-chains. However, it can also be observed that the nature of the interconnected chains gradually becomes less dense while going from Ru-30 to Ru-60. This is because of the rapid evolution of H_2_ gases and short reaction time taken at high temperatures that restricted the adjacent chain fusion by not providing sufficient time for the Ru^0^ particles to form initially. On the other hand, the inset histograms reveal a linear variation in the average chain diameter of interconnected chains and the average diameters are 3.5 ± 0.5 nm, 3.0 ± 0.2 nm and 2.6 ± 0.2 nm for Ru-30, Ru-45 and Ru-60 respectively. Fig. 2B, E and H are the HR-TEM images that show the lattice fringes and fine structures of Ru-30, Ru-45 and Ru-60 respectively. From the measured *d*-spacing values various planes are assigned. Fig. 2C, F and I are selected area electron diffraction (SAED) patterns of Ru-30, Ru-45 and Ru-60 respectively. The observed ring patterns were calibrated according to the diffraction planes from which they originate and it was found that both SAED and XRD analysis (given in ESI as Fig. S2†) are in good agreement with ICDD card 88-1734.^78^ The overall TEM, HR-TEM and SAED analyses revealed that the morphologies of Ru-30, Ru-45 and Ru-60 are interconnected nano-chain networks with differences in the densities of the network structures and the average chain diameters of the individual chains that are fused together with the adjacent chains.

### X-Ray photoelectron spectroscopic (XPS) analysis

**Fig. 3:**
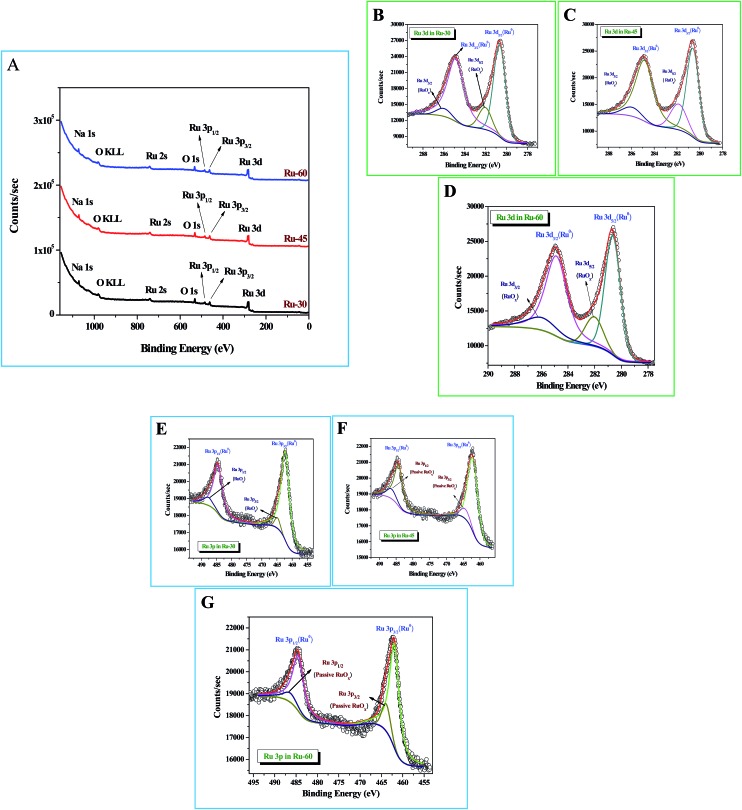
X-Ray photoelectron spectroscopic (XPS) analysis of interconnected, unprotected Ru nano-chain networks: (A) survey spectra of Ru-30, Ru-45 and Ru-60; (B–D) high resolution 3d scans of Ru-30, Ru-45 and Ru-60 respectively; (E–G) high resolution Ru 3p scans of Ru-30, Ru-45 and Ru-60 respectively.

X-Ray photoelectron spectroscopic (XPS) studies were done for Ru-30, Ru-45 and Ru-60. Fig. 3A shows the survey spectra of Ru-30, Ru-45 and Ru-60 where various peaks for Ru 3d, Ru 3p_3/2_ and Ru 3p_1/2_, Ru 2s, Na 1s and O 1s are observed at the binding energy values of 280.2 eV, 461.9 eV, 484.8 eV, 740.7 eV, 1071 eV and 529.9 eV respectively. All these peaks and their corresponding binding energy values are in agreement with the previous studies of zero-valent Ru atoms.^79^–^81^ A peak with considerable intensity for O 1s is also observed due to the spontaneous formation of a passive RuO_*x*_ film on the unprotected Ru surface when it was drawn out of the colloidal solution and dried for making XPS samples. The characteristic peak of OKLL is also observed at 980.7 eV. The peak at 1071 eV is of Na 1s from the reductant sodium borohydride. High resolution spectra of Ru 3d and Ru 3p for all three catalysts were taken and are shown in Fig. 3B–G. Fig. 3B–D are the high resolution scans of Ru 3d in Ru-30, Ru-45 and Ru-60 respectively, in which the corresponding binding energy values of Ru 3d_5/2_ and Ru 3d_3/2_ are observed at 280.50 eV and 284.9 eV for Ru-30, 280.52 eV and 284.83 eV for Ru-45 and 280.49 eV and 284.78 eV for Ru-60. It is also found that all these Ru 3d peaks have two comparatively low-intensity peaks at binding energy values of 282.1 ± 0.02 eV for Ru 3d_5/2_ and 286.06 ± 0.03 eV for Ru 3d_3/2_ which arise mainly from the passive oxide film formed on the metallic Ru surface as observed earlier because of its reactive nature in open atmosphere.^79^–^81^ Fig. 3E–G are the high resolution scans of the Ru 3p state of Ru-30, Ru-45 and Ru-60 respectively, where a doublet due to spin–orbit coupling that resulted in two states, Ru 3p_3/2_ and Ru 3p_1/2_, with a peak to peak separation of ∼22.9 eV was observed. The positions of the doublets were at 461.90 eV and 484.81 eV for Ru-30, 461.88 eV and 484.85 eV for Ru-45 and 461.87 eV and 484.77 eV for Ru-60. Like Ru 3d, each Ru 3p state also has two less intense peaks due to the passive oxide film, at 464.07 ± 0.03 eV and 486.64 ± 0.05 eV. All the observed binding energy values are nicely matching with the earlier reports of metallic Ru in many forms.^79^–^81^ From the XPS results, it is concluded that the formation of metallic Ru^0^ nanomaterials was successful under the stated conditions. Survey scans are in agreement with the EDS analyses (ESI Fig. S3A–C†) and proved that synthesized unprotected Ru^0^ nano-chains are free from foreign stabilizers.

### Mechanism for the formation of interconnected Ru nano-chain networks

The probable formation mechanism of these interconnected Ru nano-chain networks is proposed from the spectroscopic and microscopic studies carried out. The reactions occurring are:

1 RuCl_3_·*x*H_2_O → Ru^3+^ + 3Cl^–^

2 NaBH_4_ → Na^+^ + BH_4_^–^

3 BH_4_^–^ + 2H_2_O → BO_2_^–^ + 4[H]

4 Ru^3+^ + 3[H] → Ru^0^ + 3H^+^

(H_3_O^+^ + BO_2_^–^ → H_3_BO_3_ ↔ H_3_O^+^ + BO_2_^–^)

Thus the reduction of Ru^3+^ was effected in aqueous medium. The last step given inside the parentheses is the formation of boric acid and the equilibrium between the boric acid and borate oxyanion which is the conjugate base of boric acid. Depending on the pH of the medium, the concentrations of boric acid and the borate oxyanion will vary. UV-Vis spectra (Fig. 1) of the precursors and the reaction mixtures at different stages revealed that the reduction of Ru^3+^ to Ru^0^ was very fast when it was performed at high temperature (60 °C), compared to 30 °C and 45 °C. Moreover, the HR-TEM analyses revealed the interconnected nano-chain network morphology of Ru-30, Ru-45 and Ru-60. But the actual difference between the samples was in the density (density in the sense of how much closer the individual chains are in the network structures) and in the average diameters of the interconnected chains. When the synthesis was carried out at 30 °C, the rate of evolution of hydrogen was relatively slower which allowed the nucleation and growth to proceed for a longer time thereby resulting in larger individual particles (3.5 ± 0.5 nm), and the formed particles had grown along their own preferred direction and become fused with the adjacent particles that ultimately resulted in chains. These chains had further undergone cross-fusion with other chains in the vicinity and hence created denser network-like structures. Curve c of Fig. 1 shows that complete reduction of the intermediate Ru^2+^ ions by borohydride took a longer time of 30 min. The same was witnessed by visual observation also. The initial pale brown color of the Ru^2+^ ions in solutions changed to dark brown after 30 min. This was the main factor for forming larger particles and more interconnections within the network structure. When the reaction was carried out at 45 °C, the initial reduction product (Ru^2+^) took nearly 17 min to completely reduce to Ru^0^ (curve e, Fig. 1). The average chain diameter of the networks formed at 45 °C is 3.0 ± 0.2 nm, which is less than that of Ru-30. This indicates that relatively faster reduction at 45 °C decreases the growth of the particles by not providing sufficient time. However, the density of the network structure looks similar. This tells us that though the average diameter of the final chain structures is less than that for Ru-30, the time of 17 min is still more than sufficient for cross-fusion which resulted in interconnected nano-chain networks denser than Ru-30. On the other hand, when the same reaction was carried out at 60 °C, the reduction of Ru^3+^ to Ru^0^ was completed within a short time (5 min). The corresponding absorption curves are shown in Fig. 1. Curve f in Fig. 1 is the absorption spectrum of the reaction mixture (carried out at 60 °C) obtained immediately after the addition of borohydride where no peak, or only a weak peak, characteristic of the intermediate Ru^2+^ ions was observed. This indicated the immediate reduction of Ru^3+^ to Ru^0^ at higher temperature. Curve g is the absorption spectrum of the same reaction mixture 5 min after the reduction with borohydride, which in turn clearly supports the fact of immediate reduction of Ru^3+^ and the non-existence of Ru^2+^ for a long period of time, unlike in the previous cases. These observations indicate that the formation of interconnected nano-chain networks of unprotected Ru^0^ particles with smaller average diameter (2.6 ± 0.2 nm) and fewer interconnections across individual chains of the network structure requires high temperature to initiate and complete the reaction in a shorter time. Further, the smaller average chain diameter and smaller number of interconnections among the chains might be due to rapid reduction caused by the rapid evolution of hydrogen gas. As the reduction was so fast and there was no chance for the existence of intermediate Ru^2+^ ions for a significant period of time in the reaction mixture to assist the further growth of the individual particles, the reaction terminated with the smaller particles and had fewer interconnections than in Ru-30 and Ru-45. These observations clearly tell us that temperature is the main factor that took control over the average diameter and nature of the network structure.

To confirm that the observed morphology is the actual morphology in its native state too, the laser diffraction particle sizing (LDPS) technique was used. This is a technique which measures the average size of particles in their native state. In our case, it is not just the particle but it is interconnected nano-chain network. The measured average particle diameters for Ru-30, Ru-45 and Ru-60 are 721 nm, 708 nm and 632 nm respectively (Fig. S4A–C†). This clearly indicates that in solution, Ru is not present just in particle form with the same morphology as observed through TEM analysis. Moreover, it reveals that the observed morphology is not just due to evaporation phenomena on the TEM grid. If Ru was present as large particles with the sizes measured through LDPS, the same size of particle could be detected in TEM. However, that was not the case. Apart from this, we also believe that larger particles might not be transformed into an interconnected chain-like structure upon drying with an average chain diameter of 2.5 to 3.5 nm. Hence, it is clear that the synthesized colloids have the same morphology in the native state too.

### Stability of unprotected Ru^0^ nano-chain networks and its dependence on pH

**Scheme 2:**
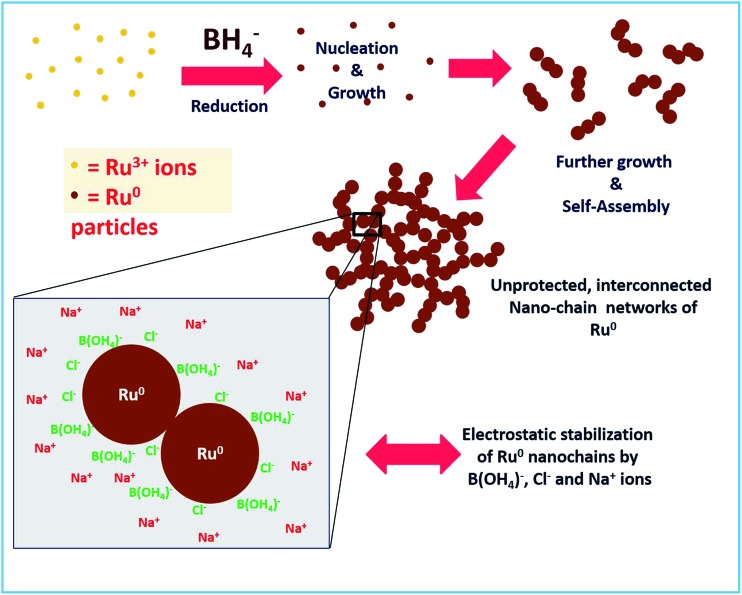
Schematic depiction of the proposed mechanism for the formation of interconnected unprotected Ru^0^ nano-chain networks and their stabilization.

The stability of a material prepared without any external stabilizing agents mainly depends on other coexisting byproducts and residual ions in solution. In the reaction mechanism section above, we proposed that boric acid and its conjugate base will be in equilibrium which depends on the pH of the solution. Besides, Na^+^ and Cl^–^ ions are also present. Hence we strongly believe that these are the actual species that stabilize the Ru particles by electrostatic interactions. Similar stabilization of Au NPs by these ions in solution was observed by Deraedt *et al.*^32^ where they reported an exceptional stabilizing activity of excess borohydride on Au NPs by the formation of hydridic bonds on the particle surface. As in our synthesis of Ru colloids, no external agent was added. Hence, obviously the only thing that is stabilizing should be the excess borohydride. However, borohydride is reactive and it will not remain in the solution after some time. Hence, at longer times, electrostatic interactions between the Cl^–^ ions and B(OH_4_)^–^ ions adsorbed on the surface of Ru NPs and the Na^+^ ions in the solution would stabilize the unprotected Ru nano-chains. The same mechanism was proposed for the stabilization of Au NPs as proposed by Deraedt *et al.*^32^ The proposed formation and stabilization mechanism are depicted in Scheme 2. We believe that the formation of the interconnected chains can also be a significant factor in the stabilizing action.

In addition, we have carried out a detailed study on the relation between the stability and the final solution pH. From Table 1, it can be seen that the final solution pH varied depending on the molar ratio of the reactants (Ru^3+^ : BH_4_^–^). The formed Ru colloidal solution is stable only when the pH is 9.7 ± 0.2, which is for a molar ratio of 1 : 1.5 (Ru^3+^ : BH_4–_). With molar ratios of 1 : 1 and 1.5 : 1, the resultant solutions are neutral (7.0 ± 0.1) and slightly acidic (6.7 ± 0.07) respectively. Here the colloidal solution remains stable for a considerable time, and the order of stability is Ru-60 > Ru-45 > Ru-30. With all other molar ratios, the resultant solutions are not as stable as the ones discussed above. The pH values of those solutions were either acidic or highly alkaline (see Table 1 for details). In these conditions, temperature had a slight effect on the stability. In acidic and highly basic conditions, at low temperature, precipitation was not effected quickly. This may be due to the slow reduction of Ru^3+^ and the existence of Ru^2+^ in solution for a considerable time. When the Ru^2+^ were reduced to Ru^0^, precipitation occurred immediately. To check the role of pH, we took 3 mL each of Ru-30, Ru-45 and Ru-60 prepared using the ratio of 1 : 1.5, and increased the pH gradually by adding 1 M NaOH drop by drop and observed that when the pH reached 10.50, precipitation began and on continued addition of NaOH, precipitation occurred with an enhanced rate. Similarly, we reduced the pH of these stable colloids by adding 0.5 M H_2_SO_4_ drop by drop and found that when the pH reached 6.60, precipitation occurred and the rate was enhanced upon continued addition. Hence, we concluded that the stability was mainly due to the byproducts and other coexisting ions that provided an appropriate electrostatic environment depending on the overall solution pH and in our present case, the optimum pH was 9.7 ± 0.2.

### Catalytic nitroarene hydrogenation

**Fig. 4:**
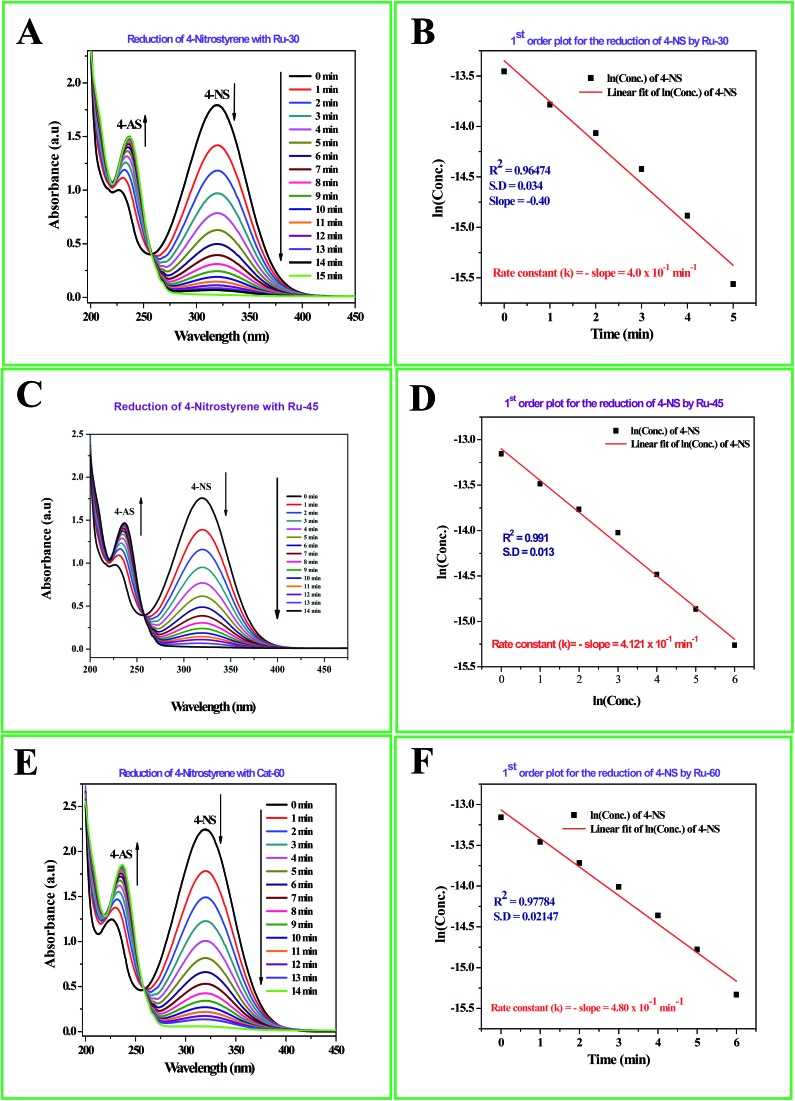
(A) Time dependent UV-Vis absorption spectra of the catalytic hydrogenation of 4-NS by Ru-30; (C) time dependent UV-Vis absorption spectra of the catalytic hydrogenation of 4-NS by Ru-45; (E) time dependent UV-Vis absorption spectra of the catalytic hydrogenation of 4-NS by Ru-60; (B), (D) and (F) are the corresponding first order plots.

**Fig. 5:**
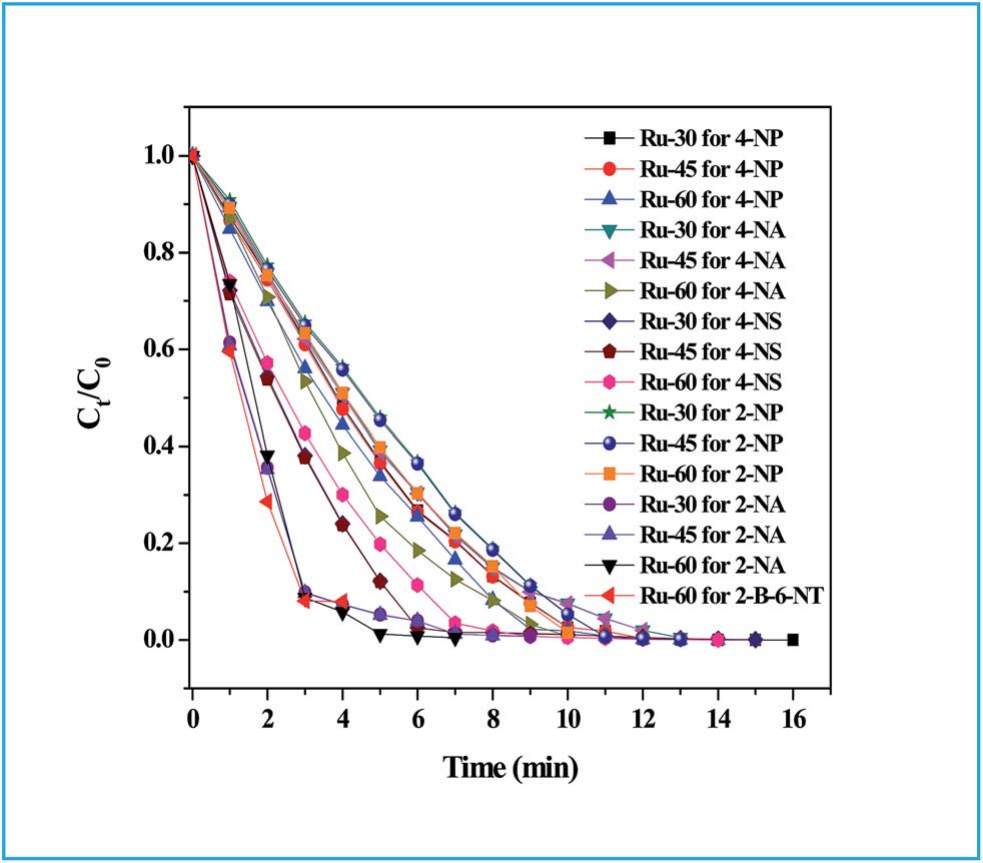
*C*_*t*_/*C*_0_*vs.* time plot of all the catalytic hydrogenation reactions by Ru-30, Ru-45 and Ru-60. Note: in all the cases the rate of disappearance of reactants when catalysed by Ru-60 is comparatively higher than Ru-30 and Ru-45.

The detailed procedure for the catalytic reduction reactions of all the nitro compounds (4-NP, 4-NA, 4-NS, 2-NP, 2-NA, 2-B-6-NT and NB) was described in the Experimental section. Nitroarene hydrogenation is the conventional and most studied catalytic reaction using zero valent metal NPs including both noble and non-noble metals.^40^,^53^,^59^ Despite being a well-studied and well documented reaction, nitroarene hydrogenation by metal NPs continues to draw great attention when it is about enhancement in the catalytic activity. Hence, any attempt to increase the catalytic rate and reduce the reaction time will be welcome. In our case we have examined the catalytic activity of our unprotected Ru^0^ catalysts (Ru-30, Ru-45 and Ru-60). Though we tried the hydrogenation of all the above nitro compounds, only 4-NP, 4-NS, 4-NA, 2-NP and 2-NA are completely reduced by Ru-30, Ru-45 and Ru-60. Interestingly, Ru-60 was able to hydrogenate 2-B-6-NT whereas Ru-30 and Ru-45 were inactive. This may be due to the larger average diameter and denser interconnected chains in Ru-30 and Ru-45 networks that restrict the coordination of 2-B-6-NT in a preferred direction to effect the hydrogenation, whereas with Ru-60 this hindrance was overcome by the smaller size and fewer interconnections. Besides the size and dense nature, we also believe that the retention of a considerable amount of Ru^2+^ ions on Ru-30 and Ru-45 surfaces may also be a key factor showing a significant restriction of this reaction. This may also be the reason for having lower rate constant values in all the reactions relative to Ru-60. However, the hydrogenation of 2-B-6-NT by Ru-60 was also not complete . None of the samples catalyzed the reduction of NB hydrogenation. Here, the catalytic reduction reaction of 4-NS is discussed in detail, and other reactions are included in ESI.† For the hydrogenation of 4-NS, the time dependent UV-Vis spectra and corresponding ln(conc.) *vs.* time plots for Ru-30, Ru-45 and Ru-60 are given as Fig. 4A–F. It can be seen that the rate constant value is the highest for Ru-60 and also the time taken by Ru-60 is shorter. All the reactions followed first order kinetics with respect to the nitro arenes and the corresponding rate constant values for Ru-30, Ru-45 and Ru-60 are 4.00 × 10^–1^ min^–1^, 4.10 × 10^–1^ min^–1^ and 4.8 × 10^–1^ min^–1^ respectively. This indicates that Ru-60 is more active than the other two samples. Similarly, the time dependent UV-Vis spectra for hydrogenation of other nitro compounds by Ru-30, Ru-45 and Ru-60 are given in ESI as Fig. S5A–M† which is in the order of 4-NP, 4-NA, 2-NP, 2-NA and 2-B-6-NT (only for Ru-60). The corresponding first order plots for Ru-30, Ru-45 and Ru-60 are also provided in the same order in ESI as Fig. S5N–Z.† Moreover, for a comparative interpretation, the catalytic activities of Ru-30, Ru-45 and Ru-60, *C*_*t*_/*C*_0_*vs.* time (*T*) are plotted for each catalytic hydrogenation reaction in Fig. 5. From Fig. 5, it is clear that the rate of disappearance of reactants in all the catalytic hydrogenation reactions was faster for Ru-60 than Ru-30 and Ru-45. Similarly, the rate of disappearance of reactants with Ru-45 is almost parallel with that for Ru-30. Beyond the time of the reaction, order and rate of the reaction, other quantitative parameters like conversion, selectivity, yield, turnover number and turnover frequency for each reaction were also calculated according to the literature reports^40^,^56^,^59^,^72^,^73^ and the detailed calculations are provided in ESI.† The results are shown in Table 2.

**Table 2:** The detailed results of catalytic hydrogenation of all the nitro compounds by Ru-30, Ru-45 and Ru-60

Reactant	Catalyst	Time (min)	Conversion (%)	Selectivity (%)	Yield (%)	TON (10^–1^)	TOF (M h^–1^)	1^st^ order *k* (×10^–1^ min^–1^)
4-NP	Ru-30	16	99	100	99	1.948	3.896	2.670
	Ru-45	15	99	100	99	1.893	3.653	2.689
	Ru-60	14	100	100	100	2.397	4.794	2.800
4-NA	Ru-30	13	99	100	99	1.348	2.023	2.900
	Ru-45	12	100	100	100	1.456	2.123	3.191
	Ru-60	10	100	100	100	1.301	2.604	3.433
4-NS	Ru-30	15	100	100	100	0.161	0.321	4.000
	Ru-45	14	100	100	100	0.169	0.33	4.121
	Ru-60	14	100	100	100	0.200	0.399	4.800
	Ru-TX-100	25	99	100	99	0.053	0.101	1.174
	Ru-CTAB	17	100	100	100	0.069	0.121	1.82
	Ru-SDS	16	100	100	100	0.055	0.136	1.838
2-NP	Ru-30	13	99	100	99	2.474	3.711	2.213
	Ru-45	13	99	100	99	2.373	3.657	2.253
	Ru-60	10	100	100	100	3.094	4.642	2.400
2-NA	Ru-30	9	99	100	99	0.120	3.600	3.010
	Ru-45	8	99	100	99	0.136	3.687	3.290
	Ru-60	7	100	100	100	1.439	4.320	3.400
2-B-6-NT	30	—	0	0	0	—	—	—
	45	—	0	0	0	—	—	—
	60	3	91.94	100	91.94	0.799	2.340	8.20

**Scheme 3:**
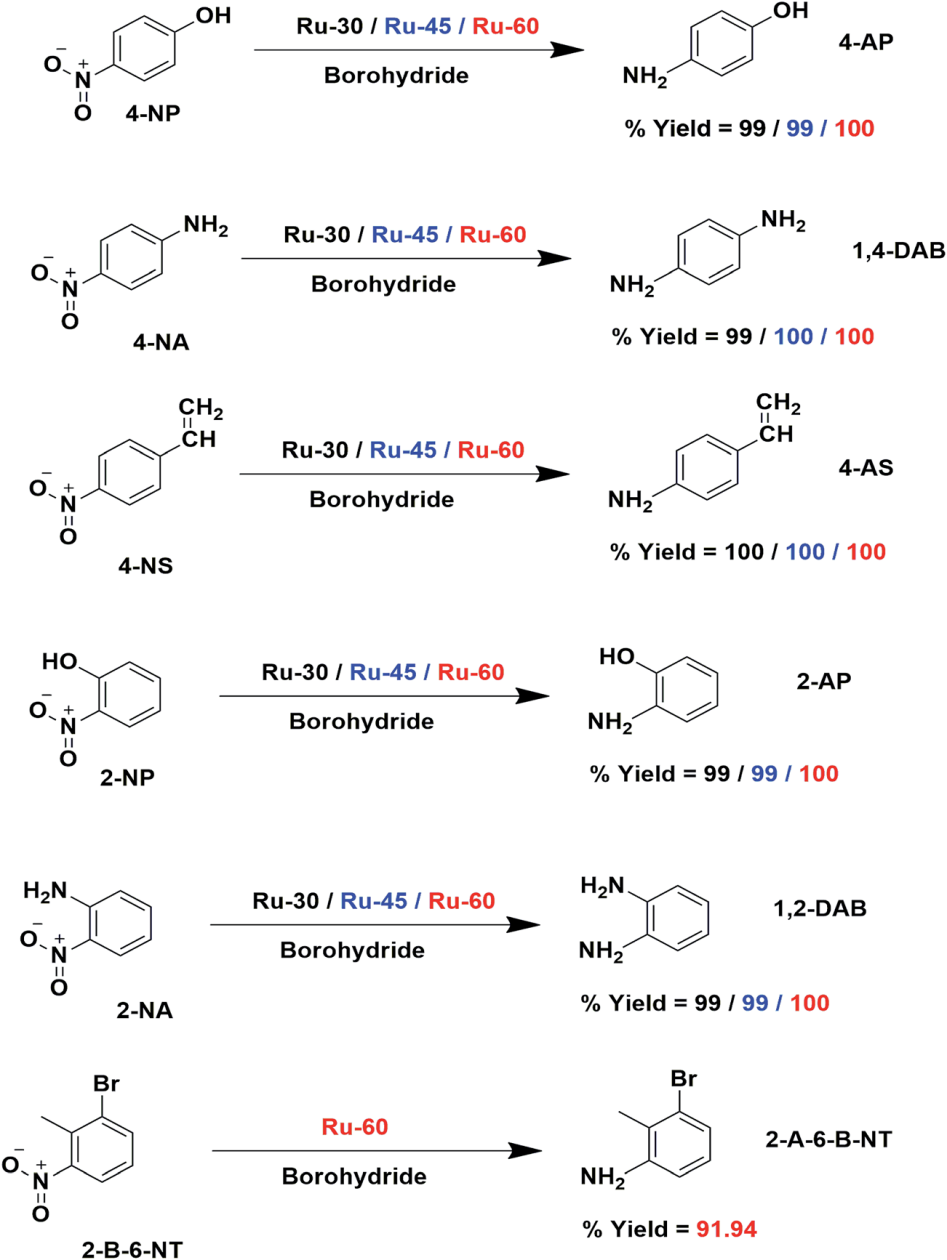
Schematic depiction of catalytic hydrogenation using Ru-30, Ru-45 and Ru-60 nanomaterials as catalysts. The catalyst and yields are shown in different colors for clarity: black indicates Ru-30, blue Ru-45 and red Ru-60.

To find reason for the superiority of our unprotected catalyst, we prepared Ru^0^ nanomaterials with three different surfactants: cetyltrimethylammonium bromide (CTAB), sodium dodecyl sulphate (SDS) and Trixon-100 (TX-100), which are cationic, anionic and neutral surfactants respectively. The formation of Ru^0^ nanomaterials with these surfactants was primarily confirmed from their absorption spectra, TEM micrographs and their electron diffraction patterns which are given as Fig. S5A–G in ESI.† We have chosen only the hydrogenation of 4-NS and studied the catalytic activities of the Ru^0^ nanomaterials under the same conditions. The resultant time-dependent UV-Vis spectra and their corresponding kinetic plots are given as Fig. S7A–F in ESI.† From these results, it can easily be evidenced that unprotected Ru^0^ catalysts are better than those capped by surfactants. The results of this comparative study are given in Table 2. We have also compared our results with other studies where the same metallic Ru NPs in other forms were used to hydrogenate nitroarenes in Table S3 in ESI.† The chosen works are closely related to the catalytic studies of this report where we found that our catalysts are comparatively better than others in terms of time and yield. However, it was observed that dendrimer encapsulated Ru NPs^73^ of smaller average individual particle size than our catalysts showed slightly higher rate constant value than ours. The much higher *k* values observed for Ru/rGO^56^ and Ru/CNF^59^ catalysts are due to the different reaction conditions employed, such as high pressure and temperature, while our reactions were performed at room temperature. The catalytic performance of our catalysts for 4-NP reduction was also compared with noble metal catalysts and other non-ruthenium catalysts as shown in Table S4 in ESI.† From these, very few studies have been reported with slightly higher rate constant values than our catalysts. Hence, we strongly believe that the unprotected surfaces of the Ru particles are the reason for the enhanced catalytic activity. The overall catalytic study revealed that Ru-60 is relatively a more active catalyst than Ru-30 and Ru-45, and all of them were significantly more active than Ru-TX-100, Ru-CTAB and Ru-SDS prepared under similar conditions. Interestingly, when compared with other Ru catalysts for the same and similar reactions, our catalysts were superior with a few exceptional cases.^59^,^73^ The overall catalytic activities of Ru-30, Ru-45 and Ru-60 are depicted in Scheme 3.

### Electrocatalytic water splitting

**Fig. 6:**
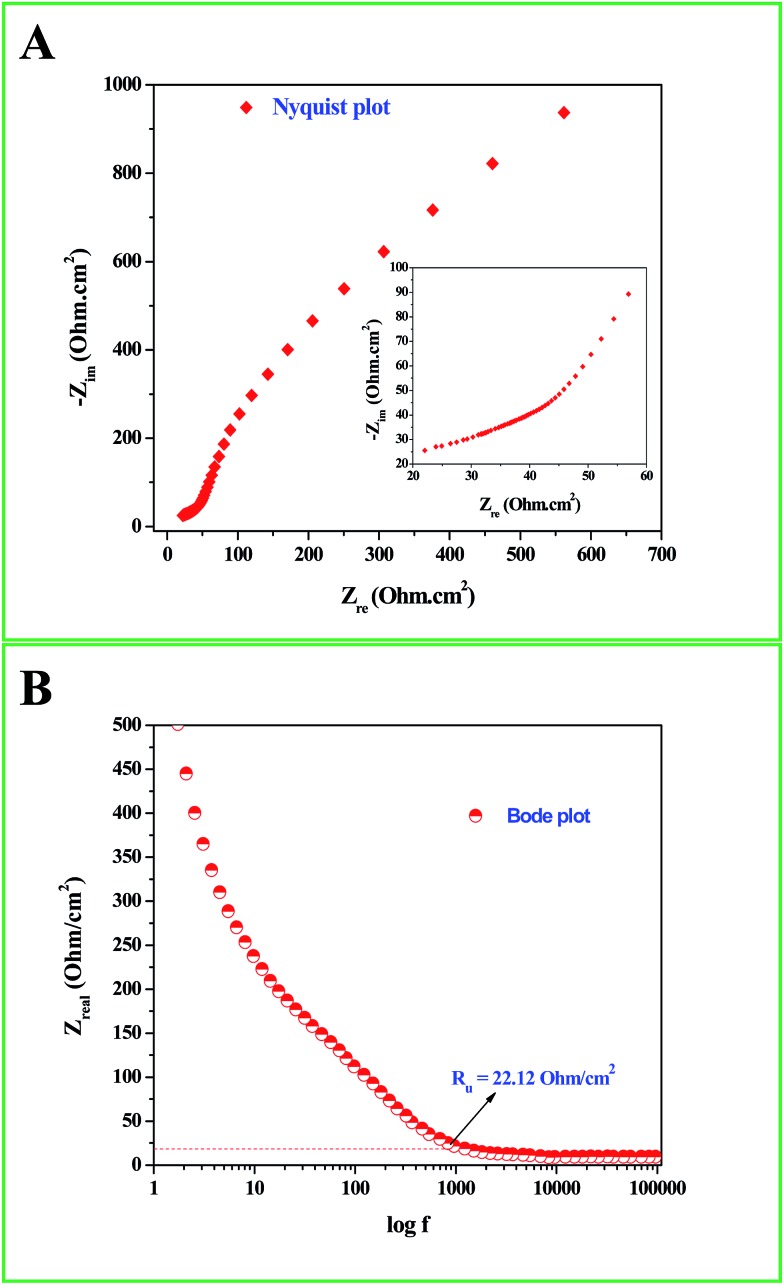
(A) Nyquist and (B) Bode impedance plots for the electrochemical system under study to determine *R*_u_.

**Fig. 7:**
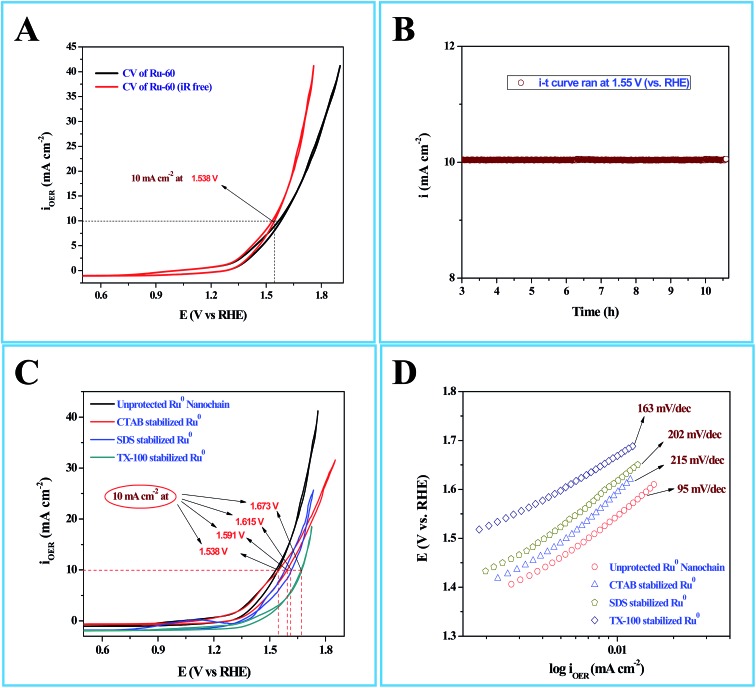
(A) Cyclic voltammogram (CV) of electrocatalytic water splitting by a Ru-60 modified GC electrode where the black curve is the CV of the with uncompensated resistance (*R*_u_), and the red curve is the CV with *iR* drop correction. (B) Chronoamperometric *i*–*t* profile for the same Ru-60 modified GC electrode. (C) CVs of electrocatalytic water splitting by Ru-60, Ru-CTAB, Ru-SDS and Ru-TX-100 modified GC electrodes. (D) The corresponding steady state polarization curves.

Interesting results obtained in the catalytic hydrogenation of nitroarenes prompted us to study the electrocatalytic performances of Ru-30, Ru-45 and Ru-60. Ru is one of the most studied transition metals along with Ir for anodic water splitting. As the need for renewable energy is receiving increased attention around the world, the need to produce H_2_ at cheaper cost has become an essential factor in the energy sector. Ru and RuO_2_ have been studied in many forms. Among the following are some important reports where Ru was used as a metal,^82^ as its oxide (RuO_2_),^83^ as its mononuclear/polynuclear complexes,^69^,^70^ as bimetallic materials^71^,^84^ and as metal oxide composite materials.^67^ Hence, it is essential to study the electrocatalytic activity of unprotected Ru-30, Ru-45 and Ru-60 catalysts for OER. As described in the experimental section, the electrocatalytic studies were carried out initially for Ru-30, Ru-45 and Ru-60 by running a cyclic voltammogram at a sweep rate of 10 mV s^–1^ in 0.1 M NaOH. The resultant CV is given as Fig. S8 in ESI.† The drop due to uncompensated resistance (*R*_u_) was corrected by carrying out electro-impedance spectroscopic analysis, and the corresponding Nyquist and Bode plots are given as Fig. 6A and B. From these plots, the *R*_u_ value found was about 22.36 ohm cm^–2^. From these figures, we can see that Ru-30, Ru-45 and Ru-60 required an overvoltage of 308 ± 2 mV to produce 10 mA cm^–2^ of OER current density. Unlike the catalysis results, similar electrocatalytic activity was observed for all the three catalysts. This can be explained as follows: the catalytic behaviour of any metallic catalyst is mainly dependent on the atoms on the surface. In our case, Ru-30 and Ru-45 were found to have considerable Ru^2+^ ions on their surfaces as evidenced by UV-Vis and XPS analyses, whereas with Ru-60 it is not the case. However, when it comes to electrocatalysis, especially anodic water splitting, the metal will undergo vigorous oxidation known as anodization. These oxides are the actual catalysts that cause the water splitting. In our case all the Ru atoms were oxidized to RuO_2_ that catalyzed the water splitting. This might be the reason for the similar electrocatalytic activity of all samples. Hence, we chose Ru-60 and performed further electrochemical analysis. Fig. 7A is the cyclic voltammogram (CV) of a Ru-60 modified GC electrode with uncompensated resistance (black curve) and *iR* free CV (red curve) run at 10 mV s^–1^ in 0.1 M NaOH. It can be seen that the benchmarking OER current density of 10 mA cm^–2^ is achieved at 1.538 V (*vs.* RHE). Hence, the overvoltage is 308 mV, which is significantly lower. Fig. 7B is the chronoamperometric curve of a Ru-60 modified GC electrode under the same experimental conditions at 1.55 V and it is observed that the modified catalyst is stable for more than 10 h.

**Fig. 8:**
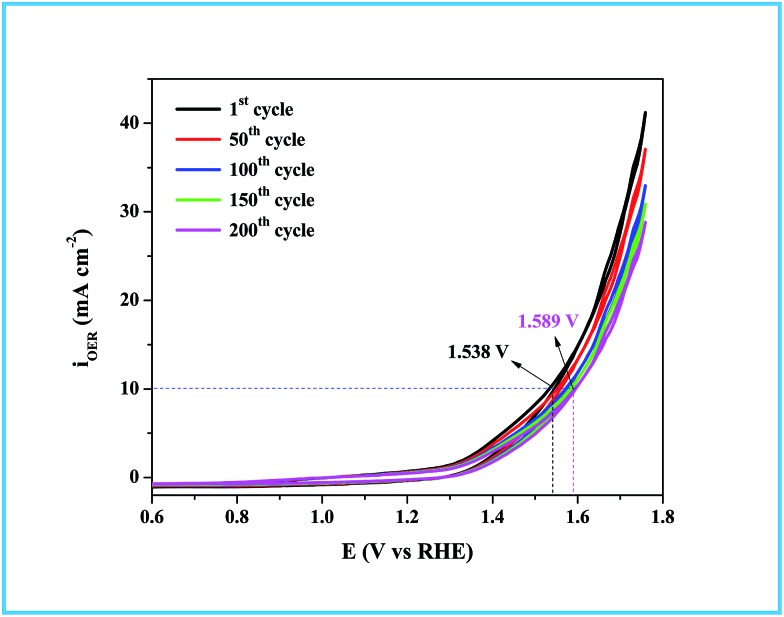
Potential sweeping test up to 200 cycles on Ru-60 modified GC at a scan rate of 10 mV s^–1^.

As in the catalysis study, we conducted a similar comparative electrocatalytic study with Ru-CTAB, Ru-SDS and Ru-TX-100 adapting the same procedure. Fig. 7C is the CVs of Ru-60 (black), Ru-CTAB (red), Ru-SDS (blue)and Ru-TX-100 (green) run at 10 mV s^–1^ in 0.1 M NaOH. The required overvoltages for an anodic current density of 10 mA cm^–2^ by these surfactant stabilized Ru^0^ nanomaterials are 361 mV, 385 mV and 441 mV for Ru-CTAB, Ru-SDS and Ru-TX-100. From this, unprotected Ru^0^ catalysts were found to be more reactive than those covered with surfactants, which in turn emphasizes the significance of unprotected surfaces in electrocatalysis. In all the cases the backward sweep was taken for the calculation of overvoltage to avoid any non-faradaic contribution to the total current and the same was done for Tafel analyses also. The corresponding steady state polarization curves for Ru-60, Ru-CTAB, Ru-SDS and Ru-TX-100 are given in Fig. 7D from which the order of the Tafel slopes were found to be 95 mV dec^–1^ for Ru-60, 215 mV dec^–1^ for Ru-CTAB, 202 mV dec^–1^ for SDS and 163 mV dec^–1^ for Ru-TX-100. This again implies that the kinetics is sluggish when the catalysts are stabilized with surfactants. The comparative electrocatalytic OER results reported in Table 3. Though the chronoamperometric analysis showed the robustness of our catalyst, we carried out post-cycling CV under the same conditions after vigorous electrolysis for 10 h, the results are given as Fig. S9 in ESI.† From Fig. S9,† a loss which was not beyond 0.05 V in terms of overpotential was observed. It may be due to leaching of catalyst in alkaline solutions which was observed previously by others for Ru catalysts in OER where the leaching product was volatile RuO_4_.^85^,^86^ Moreover, the stability of the Ru-60 modified GC was examined by potential sweeping in the same potential window at a scan rate of 10 mV s^–1^. The resultant voltammograms are given as Fig. 8 from which a steady decrease in the overall activity as well as a steady increase in the overpotential can be observed, and the increase in the overpotential is not more than 0.05 V. Constant potential electrolysis and potential sweeping caused similar changes in the electrocatalytic activity of the Ru-60 modified GC and imply its robustness under these harsh conditions.

**Table 3:** Results of the comparative OER studies on unprotected Ru nano-chain and Ru nanomaterials stabilized by TX-100, CTAB and SDS

Catalyst	*E* @ 10 mA cm^–2^ (mV)	*η* @ 10 mA cm^–2^ (mV)	Tafel (mV dec^–1^)	Specific activity (mA cm_ECSA_^–2^)	TOF @ *η* = 308 mV (s^–1^)
Unprotected Ru^0^ nano-chain networks	1538	308 ± 2	95	0.1008	4.72
Ru^0^-TX-100	1673	443	163	—	—
Ru^0^-CTAB	1591	361	215	—	—
Ru^0^-SDS	1615	385	202	—	—

**Fig. 9:**
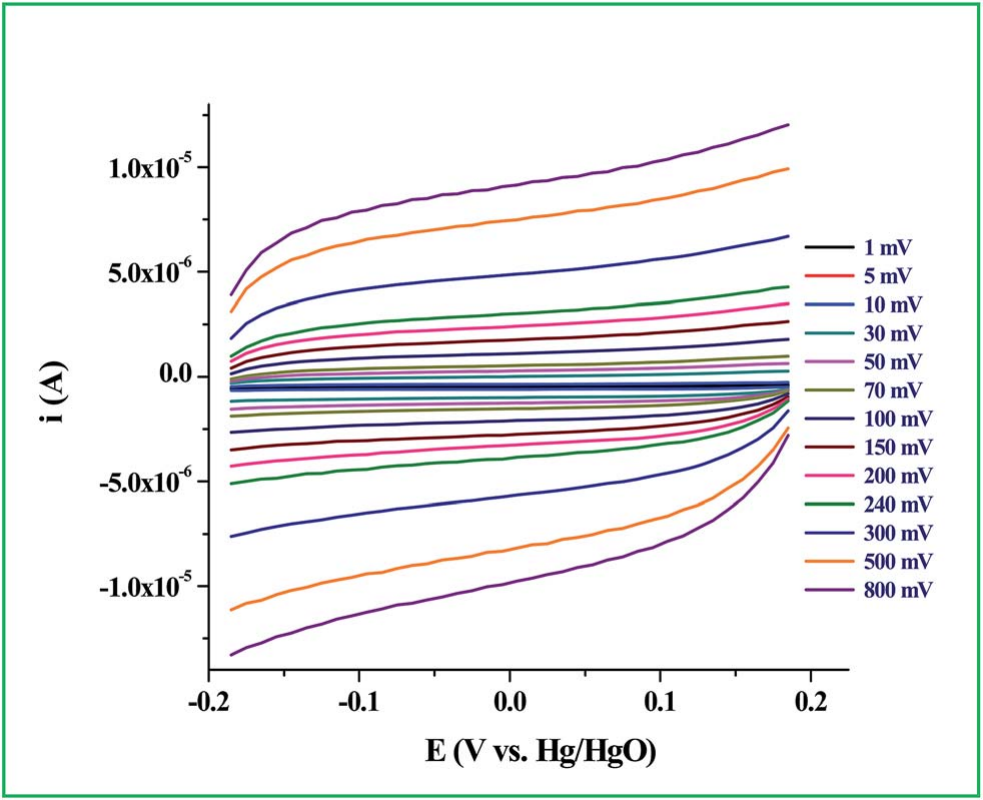
CV response of Ru-60 modified GC electrode at different scan rates.

Determination of the real surface area or the electrochemical surface area (ECSA) of the working electrode is an important parameter in an electrocatalytic reaction, which will provide quantitative information about the electrocatalytic processes. In our case, we determined the ECSA from the double layer capacitance (*C*_DL_) of the Ru-60 modified GC electrode, as described by others.^87^,^88^ The double layer charging currents (*i*_c_) were measured in the non-faradaic region from the CV curves run at different scan rates: 1 mV s^–1^, 5 mV s^–1^, 10 mV s^–1^, 30 mV s^–1^, 50 mV s^–1^, 70 mV s^–1^, 100 mV s^–1^, 150 mV s^–1^, 200 mV s^–1^, 240 mV s^–1^, 300 mV s^–1^, 500 mV s^–1^ and 800 mV s^–1^, in the same potential window. The resultant CVs are shown in Fig. 9. The double layer charging current was increased as expected due to the gradual increase in the scan rates. The relationship between the double layer charging current (*i*_c_) and the scan rate (*ν*) is given in eqn (5).

5 *i*_c_ = *νC*_DL_

**Fig. 10:**
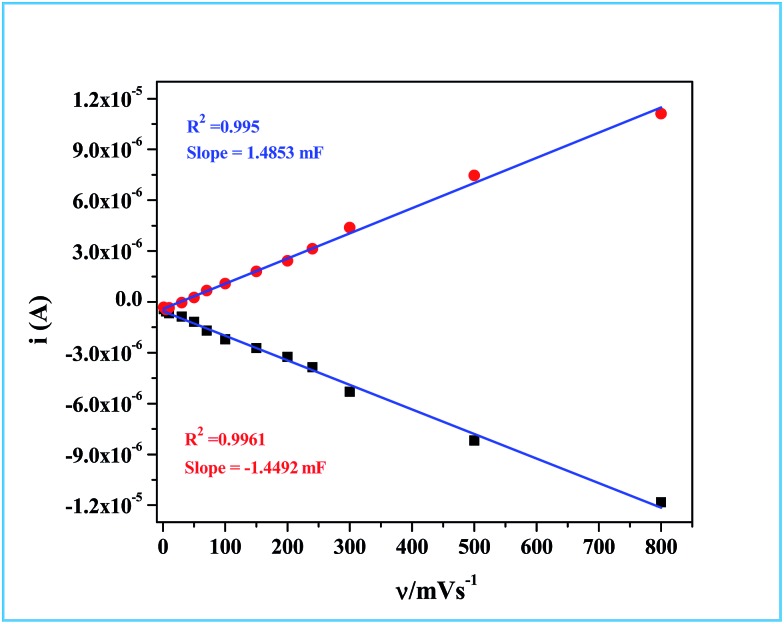
The plot of double layer charging current values obtained at different scan rates against the scan rates for determining the double layer capacitance (*C*_DL_) and ECSA.

Hence, the plot of double layer charging current (*i*_c_) against scan rate yielded a straight line, the slope of which is a direct measure of the double layer capacitance of the electrode as seen in Fig. 10. The relationship between the ECSA and the double layer capacitance (*C*_DL_) is given in eqn (6).

6 ECSA = *C*_DL_/*C*_s_

where *C*_s_ is the specific capacitance of RuO_2_. Detailed studies of the specific capacitance of RuO_2_ have been carried out by many researchers under identical experimental conditions in both alkaline and acidic media. Typically in alkaline medium RuO_2_ is reported to have specific capacitances that vary from 0.013 mF cm^–2^ to 0.019 mF cm^–2^.^89^–^95^ In our calculation we have used the specific capacitance (*C*_s_) value of 0.015 mF cm^–2^. Details of the calculation are provided in ESI.† The real or the electrochemical chemical surface area (ECSA) determined through this method for the Ru-60 modified GC electrode was 99.2 cm^2^. Based on the ECSA, the specific activity of our material was found to be 0.1008 mA cm_ECSA_^–2^ (see ESI† for detailed calculation). The observed value is higher than the report of Lee *et al.*^82^ and comparable to the report of McCrory *et al.*^96^ where they took the measured current density at 1.59 V *vs.* RHE, whereas we took the value at 1.54 V *vs.* RHE. The turnover frequency (TOF) of our catalyst was calculated by assuming Ru monolayer formation upon modifying GC with Ru-60, as we and others reported earlier for other similar noble metal electrocatalysts.^97^,^98^ The number of Ru atoms on the Ru monolayer was taken from the report of Brongersma. *et al.*^99^ Details of the TOF calculation are given in ESI.† The calculated TOF value is 4.72 s^–1^. The overall electrocatalytic study revealed that Ru-30, Ru-45 and Ru-60 had similar electrocatalytic activities in alkaline water splitting (OER) which required an overvoltage of 308 ± 2 mV to generate 10 mA cm^–2^ of OER current density with an ECSA value of 99.2 cm^2^. However, the electrocatalytic study also revealed the advantage of having an unprotected Ru catalyst from its lower overvoltage (308 ± 2 mV) required to generate a current density of 10 mA cm^–2^ and with a significant TOF value of 4.72 s^–1^. While comparing our results with other reports^67^,^69^–^71^,^82^–^84^ where Ru was used in many forms, it can be concluded that unprotected Ru surfaces showed good activity in terms of lower oxygen overpotential (10 mA cm^–2^ OER current density at 308 ± 2 mV), loading (0.205 mg cm^–2^) and TOF value (4.72 s^–1^). Although our catalysts have shown better electrocatalytic activity, there are reports with comparable activity in terms of overpotential and specific activities.^82^,^84^,^96^ Moreover, it should not be forgotten that though the synthesized material remains as Ru^0^ in solution, when it is exposed to the atmosphere the surface is oxidized to oxides RuO_*x*_ and upon anodizing, and these species are the actual catalysts of the water splitting reaction. However, in the case of surfactant-protected Ru^0^ nanomaterials, such oxidation is likely to be more difficult at lower overpotential regions which may decrease their electrocatalytic activity.

## Conclusion

In summary, three different interconnected, unprotected Ru NPs as nano-chain networks were prepared by a simple wet chemical reduction route using borohydride without any external stabilizer at three different temperatures, *viz.* 30 °C, 45 °C and 60 °C. The optimum molar ratio between Ru^3+^ and BH_4_^–^ and the pH for obtaining stable colloids are 1 : 1.5 and 9.7 ± 0.2. The temperature was increased systematically to vary the average chain diameter as 3.5 ± 0.5 nm, 3.0 ± 0.2 nm and 2.6 ± 0.2 nm for Ru-30, Ru-45 and Ru-60 respectively. Comparative and systematic studies on catalytic nitroarene hydrogenation and electrocatalytic water splitting in alkaline solution were carried out, and it was found that unprotected Ru^0^ catalysts are better catalysts than those stabilized by surfactants in both catalysis and electrocatalysis. Among Ru-30, Ru-45 and Ru-60, a significant difference in the catalytic behavior was noticed which was attributed mainly to the presence of Ru^2+^ ions on the surface of Ru-30 and Ru-45. In all the reactions, Ru-60 was found to be more active. Interestingly, all of the samples were found to show improved catalytic activity over other Ru catalysts in many forms with solid supports for the same and similar reactions, with few exceptions. The highest first order rate constants for Ru-30, Ru-45 and Ru-60 were found to be 4.0 × 10^–1^ min^–1^, 4.1 × 10^–1^ min^–1^ and 4.8 × 10^–1^ min^–1^ respectively. The unprotected Ru^0^ nano-chain networks were found to be superior to noble metal catalysts in terms of catalytic activity. Electrocatalytic anodic water splitting was catalyzed by all of them with similar efficiencies. The required overvoltage for generating the OER current density of 10 mA cm^–2^ was just 308 ± 2 mV, which is better than many other reported values for Ru catalysts. The ECSA was determined to be 99.2 cm^2^ and the TOF was 4.72 s^–1^. The specific activity is as good as very few earlier reports which is 0.1008 mA cm_ECSA_^–2^. As a consequence of the unprotected Ru^0^ nano-chain networks, superior catalytic activity and good performance in electrocatalytic water splitting were achieved. In the future, the same protocol may be extended to prepare many other metal nanomaterials with unprotected surfaces for better catalytic and electrocatalytic performances without contaminating the system by the use of any foreign stabilizing agents such as stabilizers, surfactants, supports, scaffolds and templates.

## Supplementary Material

Supplementary information
