# Supplementary material for: Unprotected and interconnected Ru0 nano-chain networks: advantages of unprotected surfaces in catalysis and electrocatalysis
Source: Chem Sci. 2016 Jan 20;7(5):3188–205. doi: 10.1039/c5sc04714e (PMC6005342; doi:10.1039/c5sc04714e)
Supplement: Supplementary file 1 [file SC-007-C5SC04714E-s001.pdf]

Electronic Supplementary Material (ESI) for Chemical Science.
This journal is © The Royal Society of Chemistry 2016

Online Supporting Information for

Unprotected and Interconnected Ru^0 Nano-chain Networks: Advantages of Unprotected Surfaces in Catalysis and Electrocatalysis

S. Anantharaj, M. Jayachandran and Subrata Kundu*

Electrochemical Materials Science (ECMS) Division, CSIR-Central Electrochemical Research Institute (CECRI), Karaikudi-630006, Tamilnadu, India.

* To whom correspondence should be addressed, *E-mail: skundu@cecri.res.in and subrata_kundu2004@yahoo.co.in,* Fax: +91 4565-227651; Tel: +91 4565-241487.

Experimental Section

Reagents.

Sodium borohydride ($NaBH_4$) and hydrated ruthenium trichloride salt ($RuCl_3.xH_2O$) were procured from Sigma-Aldrich. 4-Nitrophenol (4-NP), 4-Nitroaniline (4-NA), 4-Nitrostyrene (4-NS), 2-Nitrophenol (2-NP), 2-Nitroaniline (2-NA), 2-Bromo-6-Nitrotoluene (2-B-6-NT) and Nitrobenzene (NB) were obtained from Alfa Aesar and used without any further purification. Sodium hydroxide and concentrated sulphuric acid were purchased from SRL, India. Ag/AgCl reference electrode, Pt-foil counter electrodes and 5% nafion solution were purchased from Sigma-Aldrich. Glassy carbon (GC) working electrode of 0.0732 cm^2 area was used as working electrode after modifying with the catalysts. Milli Q water (18 MΩ) was used for the entire synthesis, catalysis and electrocatalysis processes. All the potential observed with Hg/HgO are converted to RHE scale with reference to the literature.[1,2]

Instruments.

The synthesized interconnected Ru^0 nanochain networks were characterized with several spectroscopic techniques. UV-visible (UV-Vis) absorption spectra were recorded in a double

beam UV-Vis spectrophotometer purchased from Unico (model 4802) equipped with a 1 cm quartz cuvette holder for liquid samples. The transmission electron microscopy (TEM) analysis was done with JEOL-JEM 2010 and Tecnai model TEM instrument (Tecnai™ G^2 F20, FEI) with an accelerating voltage of 200 KV.HR-TEM, (Tecnai™ G^2 TF20) working at an accelerating voltage of 200 kV. The Energy Dispersive X-ray Spectroscopy (EDS) analysis was done with the FE-SEM instrument (Oxford) with a separate EDS detector connected to that instrument. Two separate thin films of the interconnected Ru^0 nanochain networks were fabricated on glass substrates and characterized by X-ray diffraction (XRD). The XRD analysis was done with a scanning rate of 5° min^{-1} in the 2θ range 10-90° using a Bruker X-ray powder diffractometer (XRD) with Cu K_α radiation (λ = 0.154 nm). Laser diffraction particle size (LDPS) analysis was done with the LA-960 (Bruker) particle size analyzer, operating based on Mie scattering theory provided with a measurement range of 10 to 5000 nm and equipped with two laser silicon diodes of 650 nm and 405 nm respectively. X-ray photoelectron spectroscopic (XPS) analysis was performed using a Theta Probe AR-XPS system (Thermo Fisher Scientific, UK). Electrochemical analyzer CHI6084c version 12.13 was used for the entire OER and related studies. Hg/HgO reference electrode was used along with a Pt-foil counter electrode where interconnected Ru^0 nano-chain networks modified GC electrodes were used as working electrode.

Preparation of Calibration Curves for Finding the Real Concentration at Each Point of the Hydrogenation Reaction.

To study the kinetics and other necessary parameters like conversion, selectivity, yield, TON, and TOF, the actual concentrations of the reactant at each point of the reaction should be known. To find the real concentration of any substrate with a detectable and sensitive UV-Vis absorption peak with respect to the change in concentration, preparation of calibration curve of the substrate is an easy way. Calibration curves are the linear plot of the absorbance vs. concentration of the same substrate under study but with different known concentrations. As the absorbance is a concentration dependent phenomenon, any change in concentration will bring out a linear change in the absorbance too. If the concentration of the substrate solution is increased gradually, the corresponding absorbance will also increase. As a consequence of this linear variation, the plot of absorbance values against their respective concentration will also

give a linear plot with a positive slope. According to the Lambert-Beer law, the absorbance and concentration of a compound in terms of calibration curves can be related as given equation 1.

$$\text{Absorbance} = \text{Slope} \times \text{Conc.} + \text{Intercept} \quad \text{--------------- (1)}$$

or

$$\text{Conc.} = \text{Absorbance} - \text{Intercept} / \text{Slope} \quad \text{--------------- (2)}$$

By using the equation (2), the concentration of the substrate under study at any time can be found just by measuring the absorbance values. In our catalysis study, we found that except nitrobenzene all other compounds had undergone hydrogenation within our experimental time scale. Hence, it became mandatory to prepare calibration curves for all the remaining nitroarenes. The details of concentrations of each nitro compound is tabulated as Table S1. The respective UV-Vis spectra and the calibration curves are also provided in Figures S1, A-F. Using these calibration curves, the absorbance values measured from the time-dependent UV-Vis spectra of each hydrogenation reaction for both catalysts were converted into their corresponding real concentrations accordingly for further kinetic studies.

Preparation of Samples for Further Spectroscopic and Microscopic Characterizations.

The synthesized interconnected Ru^0 nano-chain networks Ru-30 and Ru-60 were characterized using UV-Vis, HR-TEM, EDS, XRD and XPS studies. The colloidal solutions of Ru-30, Ru-45 and Ru-60 were directly used for the absorption measurement in UV-Vis spectrophotometer. The same liquid solution was used for TEM sample preparation and other thin films preparation. The samples for TEM was prepared by placing a drop of the corresponding solution onto different carbon coated Cu grids followed by slow evaporation of solvent at ambient conditions. For EDS, XRD and XPS analyses, glass slides were used as substrates for thin film preparation. The slides were cleaned thoroughly in acetone and sonicated for about 30 min. The cleaned substrates were covered with Ru-30, Ru-45 and Ru-60 colloidal solutions and then dried in air. After the first layer, subsequent layers were deposited by adding more and more Ru-30, Ru-45 and Ru-60 solutions repeatedly and dried. Final samples were obtained after 10-12 depositions and then analyzed using the above techniques. For XPS analysis, Ru-30, Ru-45 and Ru-60 solutions were dried in a vacuum oven and the solid mass obtained was used.

Results and Discussion

X-Ray Diffraction (XRD) Studies.

X-ray diffraction (XRD) patterns of Ru-30, Ru-45 and Ru-60 were obtained at a scan rate of 5° per min within the range of 10° to 90°. The resultant XRD patterns are given as Figure S2. All the three catalysts gave almost similar XRD patterns with the variation in their net intensities. Diffraction analyses revealed that the Ru-30, Ru-45 and Ru-60are having the same crystal structure regardless of their synthesis temperature. It can be seen that a predominant peak at 38° and two relatively less intense peaks nearly at 45° and 83°. The observed patterns have found good agreement with the JCPDS card number # 88-1734 which corresponds to the metallic Ru having hexagonal crystal structure of primitive lattice with the space group of $P6_3/mmc$.[77,78] Other peaks corresponding to this reference JCPDS number are not detected with considerable intensity. This indicated that the formation of interconnected Ru^0 nano-chain networks preferred the growth direction majorly in these three planes. The intense peak observed at 37° is due to the diffraction from (100) plane and peaks at 45° and 83° are due to the diffractions from (101) and (112) planes. From XRD studies, the prepared interconnected Ru^0 nano-chain networks (Ru-30, Ru-45 and Ru-60) are found to be crystalline and the results are in resonance with the results obtained through HR-TEM and SAED analysis (see main text).

Energy Dispersive X-ray Spectroscopic (EDS) Analysis.

Energy dispersive X-ray spectroscopic analysis was done to check the chemical composition and purity of the prepared interconnected Ru^0 nano-chain networks (Ru-30, Ru-45 and Ru-60). EDS spectra of Ru-30, Ru-45 and Ru-60 samples are given here as Figures S3a, S3b and S3c where similar spectral features are observed. A predominant peak for Ru and with some other peaks for Ca, O and Na can be observed in the given EDS spectra. The peak of Na came from the reductant ($NaBH_4$) added during the synthesis. Peaks of Ca and O are due to the glass substrate used to fabricate the thin film. This in turn indicates the purity of the prepared interconnected Ru nano-chain networks and also confirms the absence of other foreign materials such as surfactants, stabilizers, scaffolds, templates and supports.

Determination of Conversion (X), Selectivity (S) and Yield (Y) for the catalytic hydrogenations of nitroarenes by both Ru-30, Ru-45 and Ru-60.

Other than time and rate constant of any catalytic reactions, quantitative parameters like conversion (X), selectivity (S) and yield will give better insight into the catalytic activity of the catalyst used. Conversion can be defined as the total quantity of the reacted reactant or the product(s) formed whereas selectivity of a product is defined as the fraction of the desired product formed in the reaction with other possible products. Similarly, Yield can be defined as the ratio of the product of the conversion and the selectivity of the desired product to hundred. Here, as an example we have calculated the X, S and Y for the hydrogenation of 2-Bromo-6-nitrotoluene by Ru-60 with borohydride as reductant. It should be noted that all the values of real concentrations are found by using the calibration curves made for each nitro compound as explained in the main text.

Conversion = [(Initial Conc.) – (Conc. at time 't') / Initial Conc.)] × 100

X at t = 4 min,

= [(Initial Conc.) – (Conc. at t = 4 min) / Initial Conc.)] × 100

= $[(4.4459 \times 10^{-4} - 3.5913 \times 10^{-5}) / (4.4459 \times 10^{-4})] \times 100$

X = 91.94 %

Similarly, Selectivity is given as

Selectivity = $[(nP(t) - nP(t_0)) / nR(t_0)] \times 100$

Where,

nP(t) = is the number of products formed after time 't'

$nP(t_0)$ = is the number of products in beginning (i.e., t= 0)

nR(t0) = is the number of reactants in beginning (i.e., t= 0)

As it was a homogenous reaction and the catalyst and the product had the same volume, we can relate the number of reactant/product molecules directly to their concentration.

i.e., $nC_P(t) = nP(t)$, $nC_P(t_0) = nP(t_0)$ and $nC_R(t_0) = nR(t_0)$

So,

$$S = [(4.087 \times 10^{-4} - 0)/(4.4459 \times 10^{-4})] \times 100$$

S = 100%

Note, there was no other byproduct formed.

Yield = [(% Conversion) × (% Selectivity)] / 100

= [(91.94) × (100)] / 100

Y = 91.94 %.

By adapting the same procedure the conversion, selectivity and yield of all other reactions for both catalysts were done and the final results are given in Table 1 provided in the main text.

Determination of turnover number (TON) and turnover frequency for the catalytic hydrogenations of nitroarenes by both Ru-30, Ru-45 and Ru-60.

Turnover number (TON) and turnover frequency (TOF) are the other two important quantitative parameters for any catalytic study. As seven different nitroarenes were subjected to this catalytic hydrogenation by our unprotected and interconnected Ru^0 nano-chain networks, both TON and TOF have become the essential quantities when comparing their catalytic activity between them and among all other existing catalyst for the same and similar type of nitroarenes hydrogenation. TON can be simply defined as the ratio of the number of reactants reacted to number particles catalyzed. Hence, it just a number and gives a semi-quantitative information. On the other hand, TOF is a quantitative measure which gives the details on the number of reactants reacted per number of particles in unit time. Since the catalytic study was homogeneous and the volume of both reactants/products and the catalyst are having the same volume, we can take the number of reactants to be equal to the concentration of the same. It is applicable for the catalyst too.

TON =n(product) / n(catalyst)

We know that,

$$C = n / V$$

Since both product and catalyst are in the same reaction vessel the volume for both is the same:

$$n(\text{product}) = c(\text{product}) / V$$

$$n(\text{catalyst}) = c(\text{catalyst}) / V$$

V, the volume can be canceled down:

$$\mathbf{TON} = n(\text{product}) / n(\text{catalyst})$$

$$= [c(\text{product}) / V] / [c(\text{catalyst}) / V]$$

$$\mathbf{TON} = c(\text{product}) / c(\text{catalyst})$$

Following this expression, TON of all the catalytic reactions were calculated and listed in the Table 1 provided in the main text. To find out the real concentration of product the same calibration curves are used one again. Similarly, one can simply calculate TOF values for a homogeneous reaction as follows.

$$\mathbf{TOF} = [n\ (\text{Product}) / n\ (\text{Catalyst})] / \text{unit time}$$

$$\mathbf{TOF} = \text{TON/ unit time}$$

We can see here that the TOF of any reaction is just the ratio of TON per unit time. In our case, as used in general, we have given our TOF values calculated using the expression with the unit of mol h^{-1}.

Determination of TOF value for the electrocatalytic water splitting.

Turnover frequency for an electrocatalytic water splitting reactions can be done in more than one way, in our case we have opted the method of using OER current at particular over voltage and the surface concentration of Ru atoms at the modified GC working electrode which was calculated from the amount catalyst we added to modify the GC electrode. The corresponding expression is,

$$\mathbf{TOF} = \boldsymbol{i} \times \mathbf{N_A} / \mathbf{A} \times \mathbf{F} \times \mathbf{n} \times \boldsymbol{\Gamma}$$

Where,

i = current

N_A= Avogadro number

A = Geometrical surface area of the electrode

F = Faraday constant

n = Number of electrons

г = Surface concentration

We have taken the OER current of 10 mA/cm^2 observed at 1.538 V (vs. RHE) and calculated the TOF as follows. The number Ru atom in a monolayer of Ru atoms is 3.3×10^{15} (see ref.98 in main text)

Hence we,

$$\mathbf{TOF_{1.56V}} = [(10\times 10^{-3})\ (6.023\times 10^{23})] / [(1)\ (96485)\ (4)\ (3.3 \times 10^{15})]$$

$$\mathbf{TOF_{1.56V}} = 4.72\ s^{-1}$$

TOF calculation is being done in many methods throughout the globe in the scientific community where they differ mainly in the way by which the surface concentration of the atoms is determined. Among them, the method which uses the electrochemical CV technique is adaptable for the catalysts with distinct redox behavior that can be distinguished easily from the non-faradaic current contribution. The method based on unit-cell and surface area (BET) and assumption of monolayer formation and subsequent determination of number of atoms is suitable for other systems. Here in our study we have calculated the TOF value on the basis of monolayer assumption.

Determination of Specific Activity:

Specific activity of Ru-60 modified GC was calculated using following equation

Specific activity = $j_{1.54V}$ at 10 $mAcm^{-2} \times 1\ cm^2_{geo}$ / 99.2 cm^2_{ECSA}

= 0.1008 $mAcm^{-2}_{ECSA}$

Synthesis of Ru Nanomaterials using Surfactannts.

To extensively study the advantageous of the unprotected Ru^0 catalysts, we have synthesized Ru nanomaterials with three different surfactants such as CTAB, SDS and TX-100 by keeping all other conditions the same. The primary UV-Vis spectra (Figure S5A) revealed the successful formation Ru nanomaterials. The same were subjected TEM (Figure S5, B-D) and SAED (Figure 5, E-G) analyses which in turn confirmed the formation of Ru nanomaterials. Presence of expected diffraction planes corresponding to the zero valent Ru metal nanomaterials in SAED pattern are calibrated and identified that further confirmed the successful formation.

Figure Captions:

Figure S1: (A) is the calibration curve of 4-NP made to find the real concentration of 4-NP from its Abs. value. (B) is the calibration curve of 4-NA made to find the real concentration of 4-NA from its Abs. value. (C) is the calibration curve of 4-NS made to find the real concentration of 4-NSNP from its Abs. value. (D) is the calibration curve of 2-NP made to find the real concentration of 2-NP from its Abs. value. (E) is the calibration curve of 2-NA made to find the real concentration of 2-NA from its Abs. value. (F) is the calibration curve of 2-B-6-NT made to find the real concentration of 2-B-6-NT from its Abs. value.

Figure S2: X-ray diffraction patterns of Ru-30, Ru-45 and Ru-60.

Figure S3: Energy dispersive electronic spectra (EDS) of Ru-30 (S3A), Ru-45(S3B) and Ru-60(S3C).

Figure S4: (A-C) Average particle size histogram of Ru-30, Ru-45 and Ru-60.

Figure S5: (A-C) time-dependent UV-Vis spectra for the reduction of 4-NP by Ru-30, Ru-45 and Ru-60 respectively. (D-F) are the time-dependent UV-Vis spectra for the reduction of 4-NA by Ru-30, Ru-45 and Ru-60 respectively. (G-I) are the time-dependent UV-Vis spectra for the reduction of 2-NP by Ru-30, Ru-45 and Ru-60 respectively. (J-L) are the time-dependent UV-Vis spectra for the reduction of 2-NA by Ru-30, Ru-45 and Ru-60 respectively. (M) is the time-dependent UV-Vis spectra for the reduction of 2-B-6-NT Ru-60.(N-P) are the first order kinetics plot for the reduction of 4-NP by Ru-30, Ru-45 and Ru-60 respectively. (Q-S) are the first order kinetics plot for the reduction of 4-NA by Ru-30, Ru-45 and Ru-60 respectively. (T-V) are the first order kinetics plot for the reduction of 2-NP by Ru-30, Ru-45 and Ru-60 respectively.(W-Y) are the first order kinetics plot for the reduction of 2-NA by Ru-30, Ru-45 and Ru-60 respectively. (Z) is the first order kinetics plot for the reduction of 2-B-6-NT by Ru-60.

Figure S6: (A-C) the UV-Vis spectra of various reaction mixture encountered during the synthesis of Ru-TX-100, Ru-CTAB and Ru-SDS. (D & E), (F & G) and (H & I) are the TEM micrographs and their corresponding SAED patterns of Ru-TX-100, Ru-CTAB and Ru-SDS.

Figure S7: (A & B), (C & D) and (E & F) are the time-dependent UV-Vis spectra and their associated first order plots for the reduction of 4-NS by Ru-TX-100, Ru-CTAB and Ru-SDS respectively.

Figure S8: Cyclic voltammogram (CV) (iR_{free}) of both Ru-30, Ru-45 and Ru-60 ran at 10 mV/s.

Figure S9: Post-cycle cyclic voltammogram (CV) (iR_{free}) analysis of Ru-60 where curve a, denotes the pre-cycle LSV and b is post cycle LSV.

Table S1: Concentration of nitro compounds used to make calibration curves

Table S2: Detailed concentration and other reaction parameters of comparative catalytic study.

Table S3: Benchmarking unprotected Ru nano-chain networks as catalyst among other Ru catalysts.

Table S4: Benchmarking unprotected Ru nano-chain networks as catalyst among other noble and non-noble metal catalysts for the reduction of 4-nitrophenol by rate constant values.

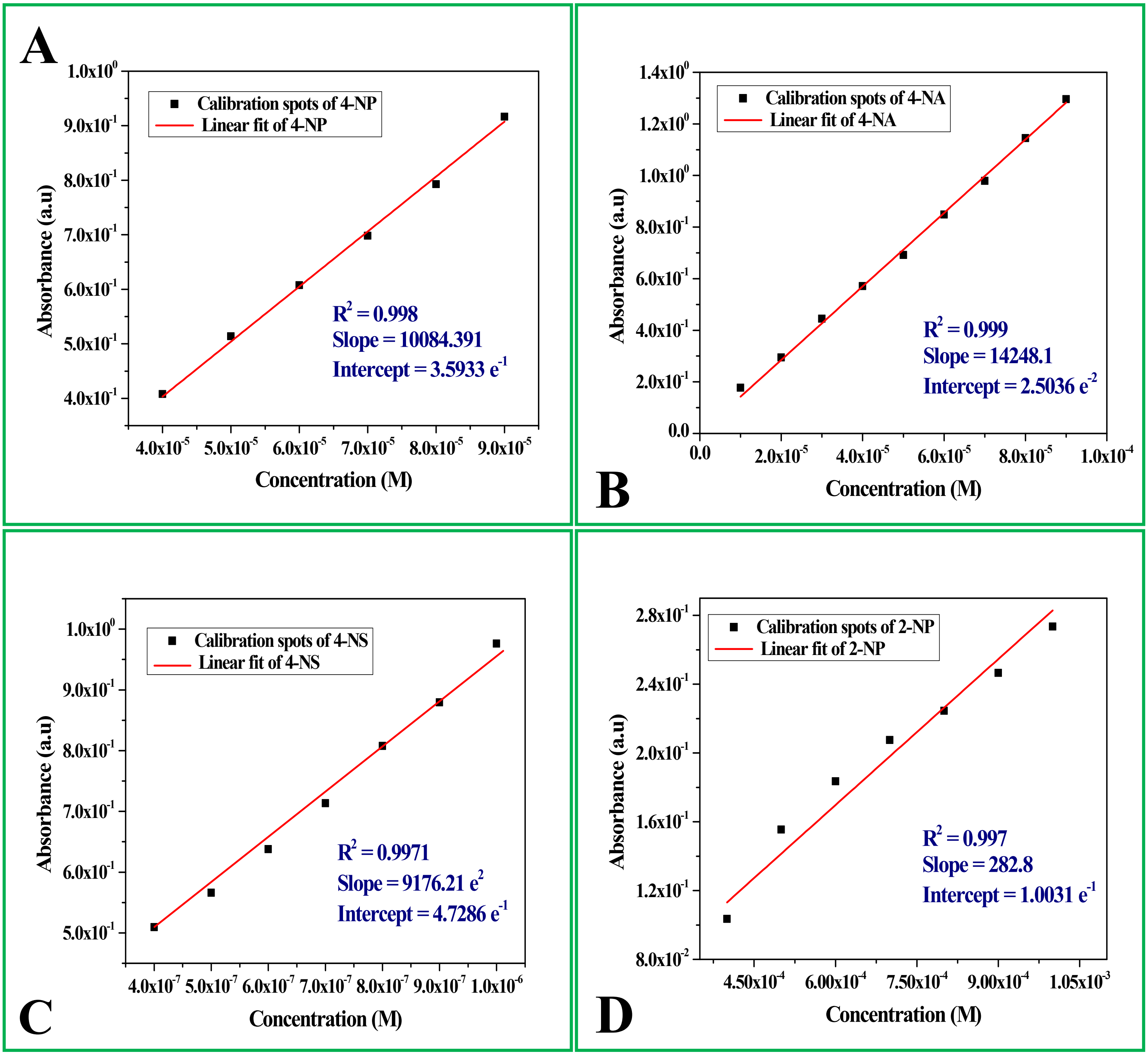

A
Calibration spots of 4-NP
Linear fit of 4-NP
R^2 = 0.998
Slope = 10084.391
Intercept = 3.5933 e^{-1}
Absorbance (a.u)
Concentration (M)
B
Calibration spots of 4-NA
Linear fit of 4-NA
R^2 = 0.999
Slope = 14248.1
Intercept = 2.5036 e^{-2}
Absorbance (a.u)
Concentration (M)
C
Calibration spots of 4-NS
Linear fit of 4-NS
R^2 = 0.9971
Slope = 9176.21 e^{2}
Intercept = 4.7286 e^{-1}
Absorbance (a.u)
Concentration (M)
D
Calibration spots of 2-NP
Linear fit of 2-NP
R^2 = 0.997
Slope = 282.8
Intercept = 1.0031 e^{-1}
Absorbance (a.u)
Concentration (M)

Figure S1, A-B

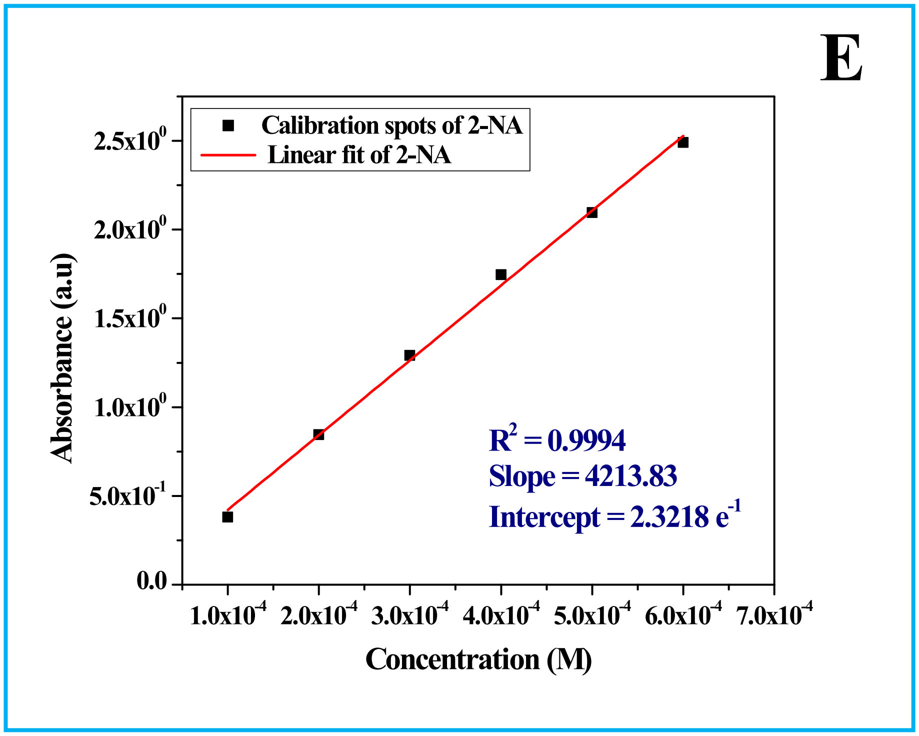
E
Calibration spots of 2-NA
Linear fit of 2-NA
$R^2 = 0.9994$
Slope = 4213.83
Intercept = 2.3218 e^{-1}
Absorbance (a.u)
Concentration (M)

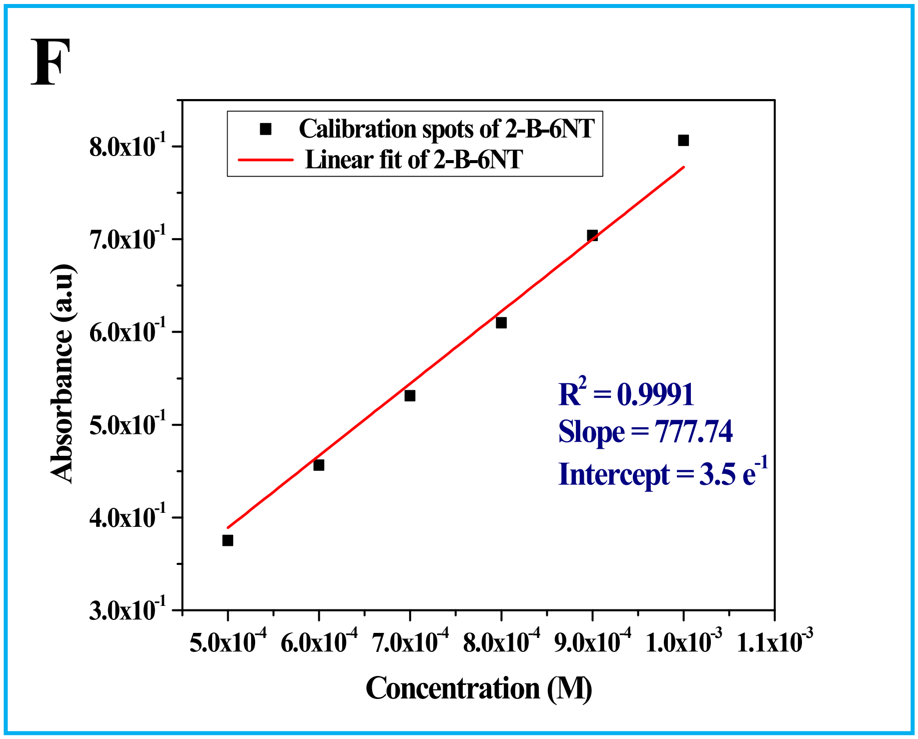
F
Calibration spots of 2-B-6NT
Linear fit of 2-B-6NT
$R^2 = 0.9991$
Slope = 777.74
Intercept = 3.5 e^{-1}
Absorbance (a.u)
Concentration (M)

Figure S1, E-F

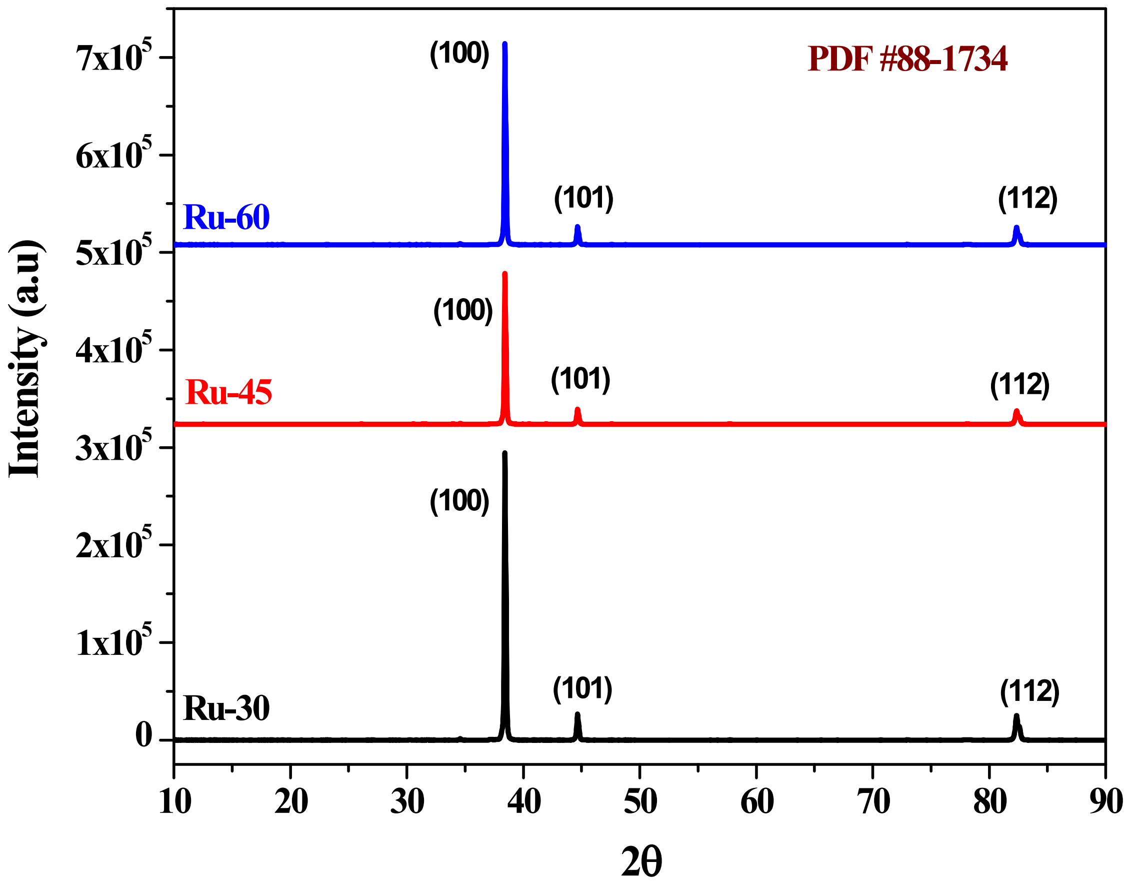
PDF #88-1734
Ru-60
Ru-45
Ru-30
(100)
(101)
(112)
Intensity (a.u)
2θ
7x10^5
6x10^5
5x10^5
4x10^5
3x10^5
2x10^5
1x10^5
0
10
20
30
40
50
60
70
80
90

Figure S2

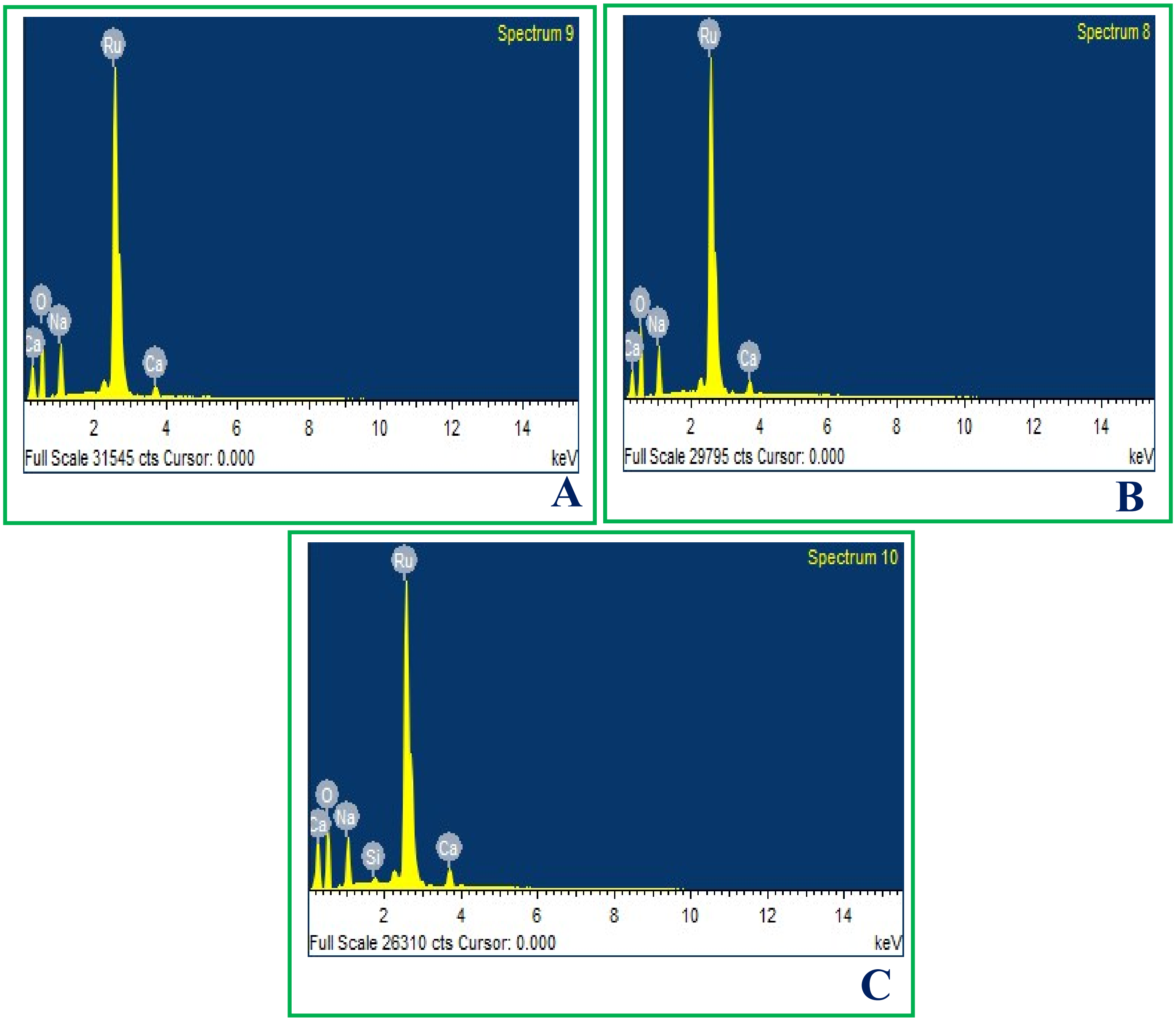
Spectrum 9
Ru
O
Na
Ca
Ca
2
4
6
8
10
12
14
Full Scale 31545 cts Cursor: 0.000
keV
A
Spectrum 8
Ru
O
Na
Ca
Ca
2
4
6
8
10
12
14
Full Scale 29795 cts Cursor: 0.000
keV
B
Spectrum 10
Ru
O
Ca
Na
Si
Ca
2
4
6
8
10
12
14
Full Scale 26310 cts Cursor: 0.000
keV
C

Figure S3, A-C

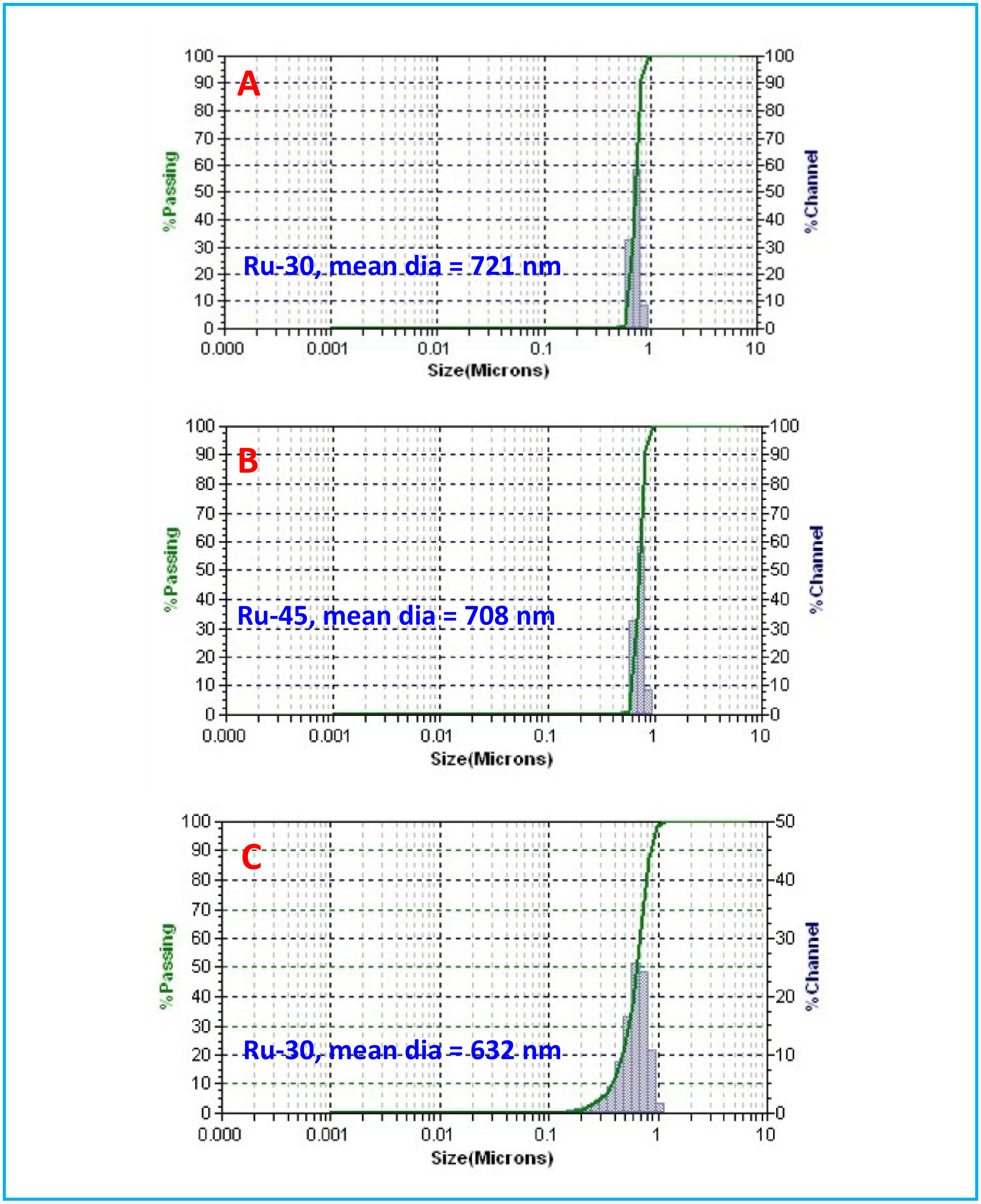
A
Ru-30, mean dia = 721 nm
B
Ru-45, mean dia = 708 nm
C
Ru-30, mean dia = 632 nm
% Passing
% Channel
Size(Microns)
0.000
0.001
0.01
0.1
1
10

Figure S4, A-C

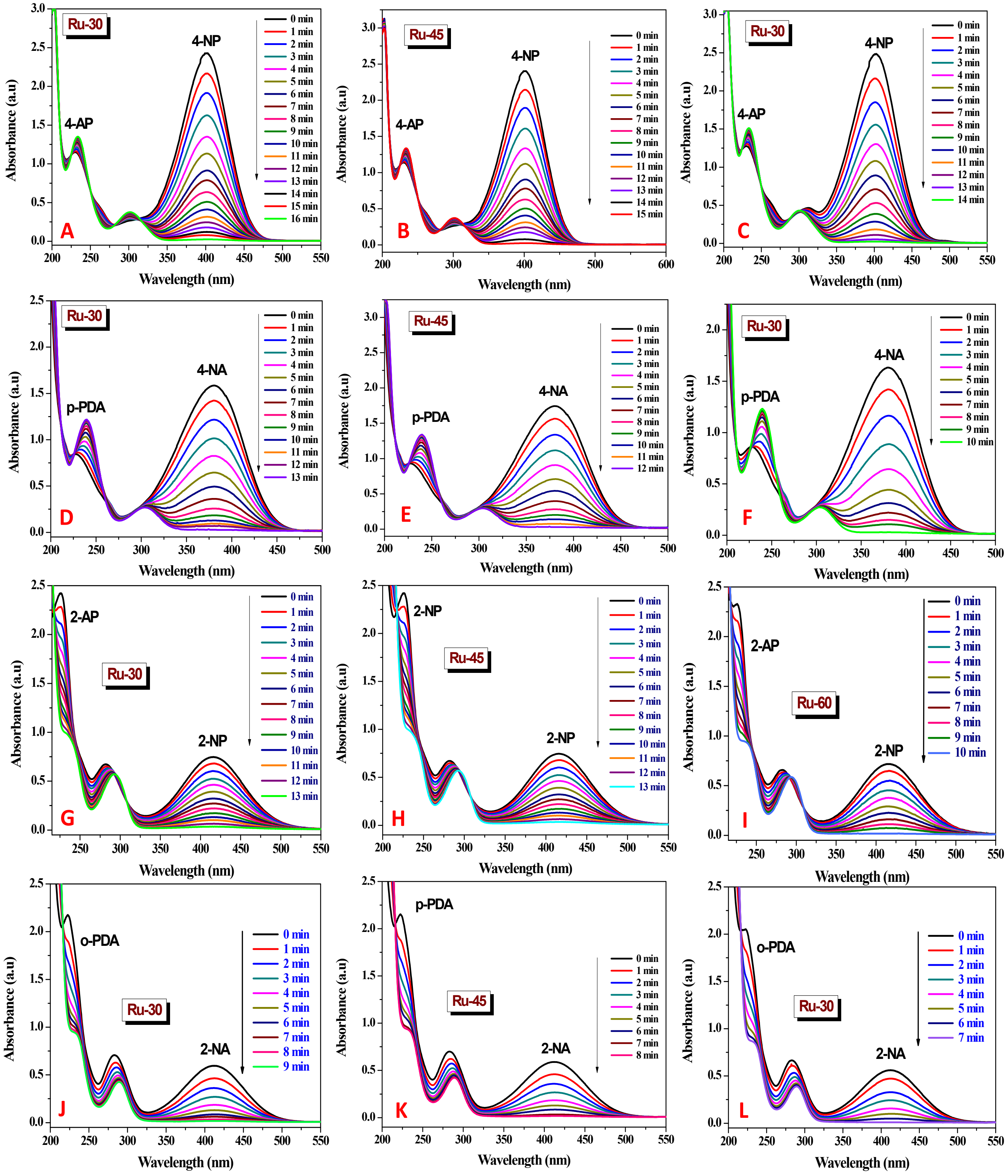
Ru-30
4-NP
4-AP
A
Ru-45
4-NP
4-AP
B
Ru-30
4-NP
4-AP
C
Ru-30
4-NA
p-PDA
D
Ru-45
4-NA
p-PDA
E
Ru-30
4-NA
p-PDA
F
2-AP
Ru-30
2-NP
G
2-NP
Ru-45
2-NP
H
2-AP
Ru-60
2-NP
I
o-PDA
Ru-30
2-NA
J
p-PDA
Ru-45
2-NA
K
o-PDA
Ru-30
2-NA
L
Absorbance (a.u)
Wavelength (nm)

Figure S5, A-L

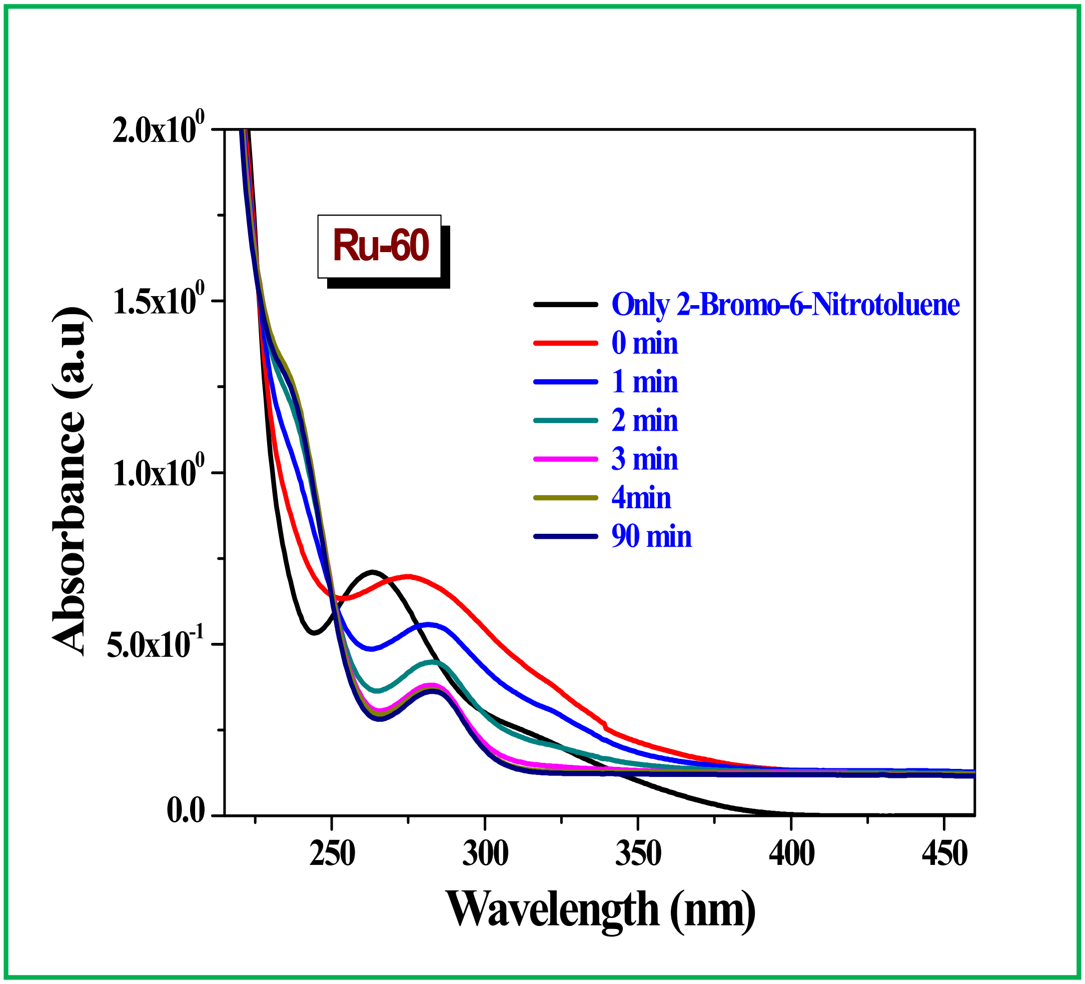

Ru-60
Only 2-Bromo-6-Nitrotoluene
0 min
1 min
2 min
3 min
4min
90 min
2.0x10^0
1.5x10^0
1.0x10^0
5.0x10^-1
0.0
250
300
350
400
450
Absorbance (a.u)
Wavelength (nm)

Figure S5, M

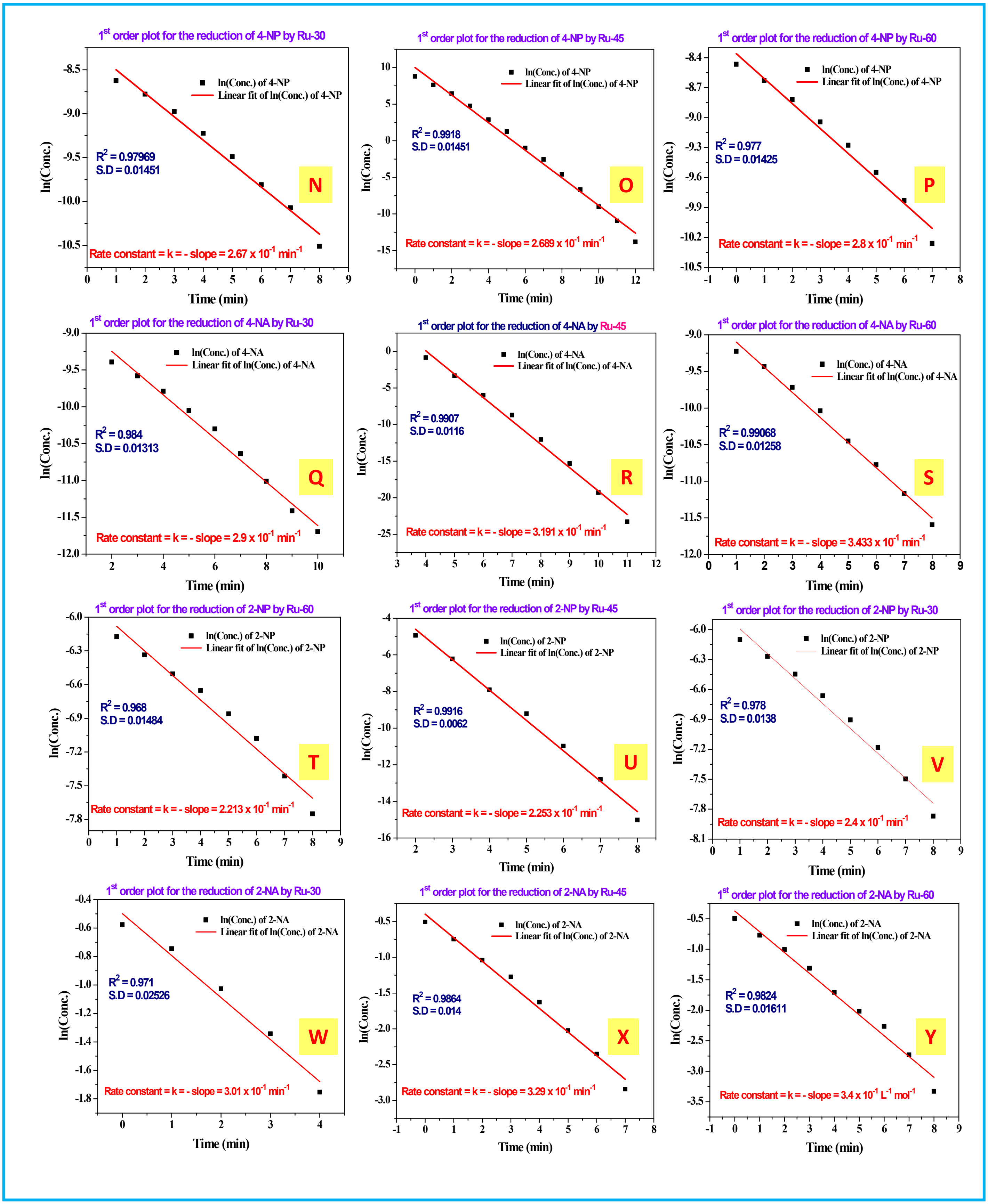

1st order plot for the reduction of 4-NP by Ru-30
ln(Conc.) of 4-NP
Linear fit of ln(Conc.) of 4-NP
R2 = 0.97969
S.D = 0.01451
N
Rate constant = k = - slope = 2.67 x 10-1 min-1
ln(Conc.)
Time (min)
1st order plot for the reduction of 4-NP by Ru-45
ln(Conc.) of 4-NP
Linear fit of ln(Conc.) of 4-NP
R2 = 0.9918
S.D = 0.01451
O
Rate constant = k = - slope = 2.689 x 10-1 min-1
ln(Conc.)
Time (min)
1st order plot for the reduction of 4-NP by Ru-60
ln(Conc.) of 4-NP
Linear fit of ln(Conc.) of 4-NP
R2 = 0.977
S.D = 0.01425
P
Rate constant = k = - slope = 2.8 x 10-1 min-1
ln(Conc.)
Time (min)
1st order plot for the reduction of 4-NA by Ru-30
ln(Conc.) of 4-NA
Linear fit of ln(Conc.) of 4-NA
R2 = 0.984
S.D = 0.01313
Q
Rate constant = k = - slope = 2.9 x 10-1 min-1
ln(Conc.)
Time (min)
1st order plot for the reduction of 4-NA by Ru-45
ln(Conc.) of 4-NA
Linear fit of ln(Conc.) of 4-NA
R2 = 0.9907
S.D = 0.0116
R
Rate constant = k = - slope = 3.191 x 10-1 min-1
ln(Conc.)
Time (min)
1st order plot for the reduction of 4-NA by Ru-60
ln(Conc.) of 4-NA
Linear fit of ln(Conc.) of 4-NA
R2 = 0.99068
S.D = 0.01258
S
Rate constant = k = - slope = 3.433 x 10-1 min-1
ln(Conc.)
Time (min)
1st order plot for the reduction of 2-NP by Ru-60
ln(Conc.) of 2-NP
Linear fit of ln(Conc.) of 2-NP
R2 = 0.968
S.D = 0.01484
T
Rate constant = k = - slope = 2.213 x 10-1 min-1
ln(Conc.)
Time (min)
1st order plot for the reduction of 2-NP by Ru-45
ln(Conc.) of 2-NP
Linear fit of ln(Conc.) of 2-NP
R2 = 0.9916
S.D = 0.0062
U
Rate constant = k = - slope = 2.253 x 10-1 min-1
ln(Conc.)
Time (min)
1st order plot for the reduction of 2-NP by Ru-30
ln(Conc.) of 2-NP
Linear fit of ln(Conc.) of 2-NP
R2 = 0.978
S.D = 0.0138
V
Rate constant = k = - slope = 2.4 x 10-1 min-1
ln(Conc.)
Time (min)
1st order plot for the reduction of 2-NA by Ru-30
ln(Conc.) of 2-NA
Linear fit of ln(Conc.) of 2-NA
R2 = 0.971
S.D = 0.02526
W
Rate constant = k = - slope = 3.01 x 10-1 min-1
ln(Conc.)
Time (min)
1st order plot for the reduction of 2-NA by Ru-45
ln(Conc.) of 2-NA
Linear fit of ln(Conc.) of 2-NA
R2 = 0.9864
S.D = 0.014
X
Rate constant = k = - slope = 3.29 x 10-1 min-1
ln(Conc.)
Time (min)
1st order plot for the reduction of 2-NA by Ru-60
ln(Conc.) of 2-NA
Linear fit of ln(Conc.) of 2-NA
R2 = 0.9824
S.D = 0.01611
Y
Rate constant = k = - slope = 3.4 x 10-1 L-1 mol-1
ln(Conc.)
Time (min)

Figure S5, N-Y

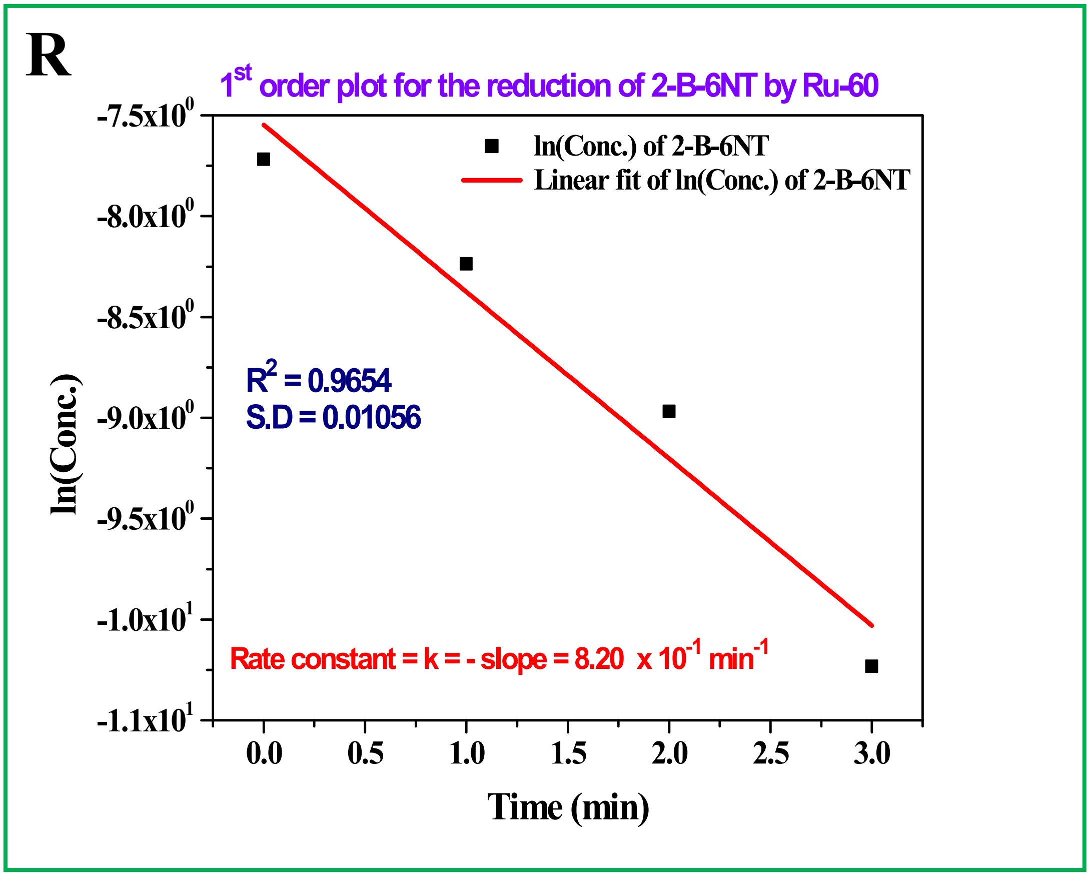
R
1st order plot for the reduction of 2-B-6NT by Ru-60
-7.5x10^0
-8.0x10^0
-8.5x10^0
-9.0x10^0
-9.5x10^0
-1.0x10^1
-1.1x10^1
ln(Conc.)
ln(Conc.) of 2-B-6NT
Linear fit of ln(Conc.) of 2-B-6NT
R^2 = 0.9654
S.D = 0.01056
Rate constant = k = - slope = 8.20 x 10^-1 min^-1
0.0
0.5
1.0
1.5
2.0
2.5
3.0
Time (min)

Figure S5, Z

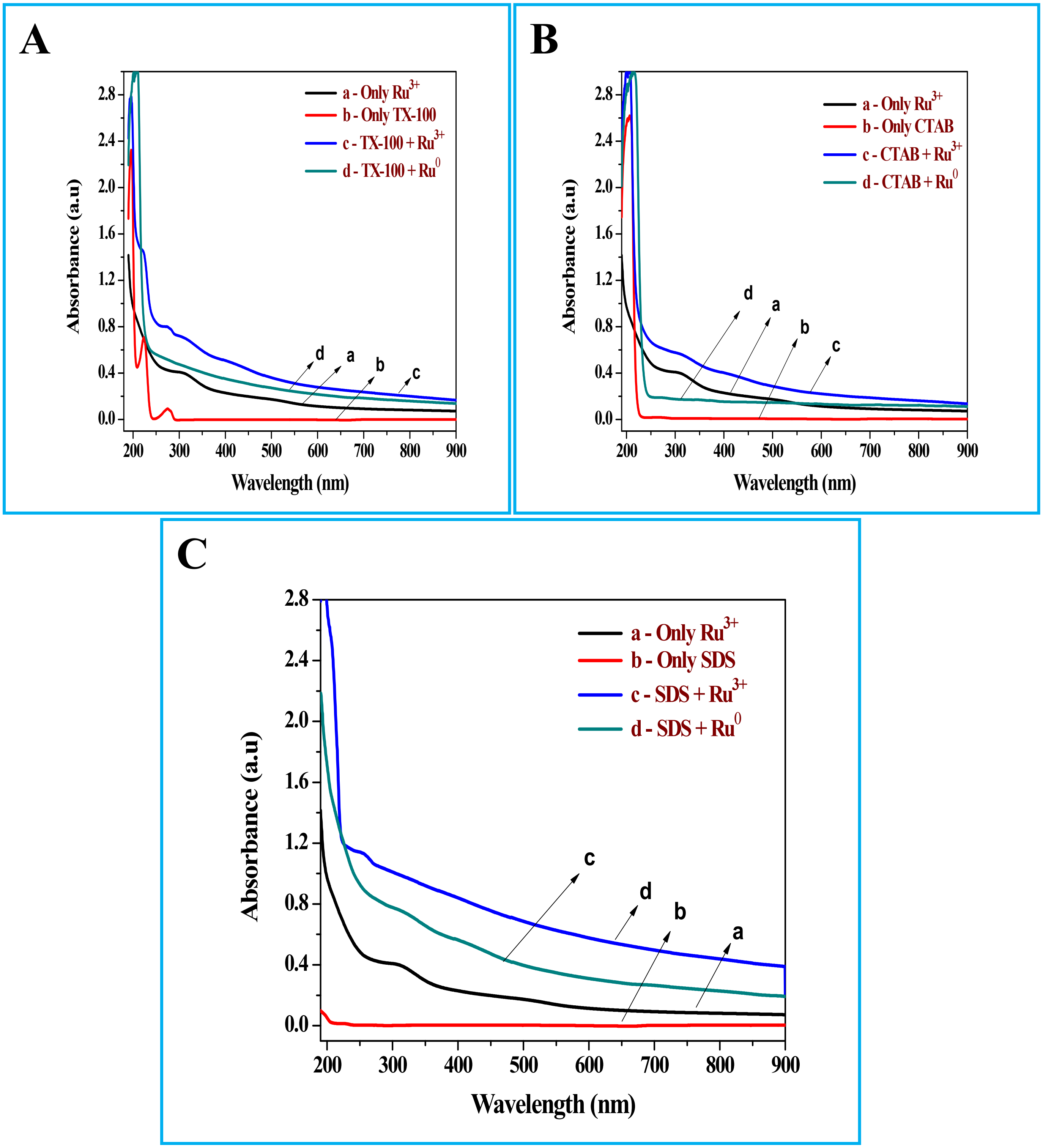
A
a - Only Ru3+
b - Only TX-100
c - TX-100 + Ru3+
d - TX-100 + Ru0
Absorbance (a.u)
Wavelength (nm)
B
a - Only Ru3+
b - Only CTAB
c - CTAB + Ru3+
d - CTAB + Ru0
Absorbance (a.u)
Wavelength (nm)
C
a - Only Ru3+
b - Only SDS
c - SDS + Ru3+
d - SDS + Ru0
Absorbance (a.u)
Wavelength (nm)

Figure S6, A-C

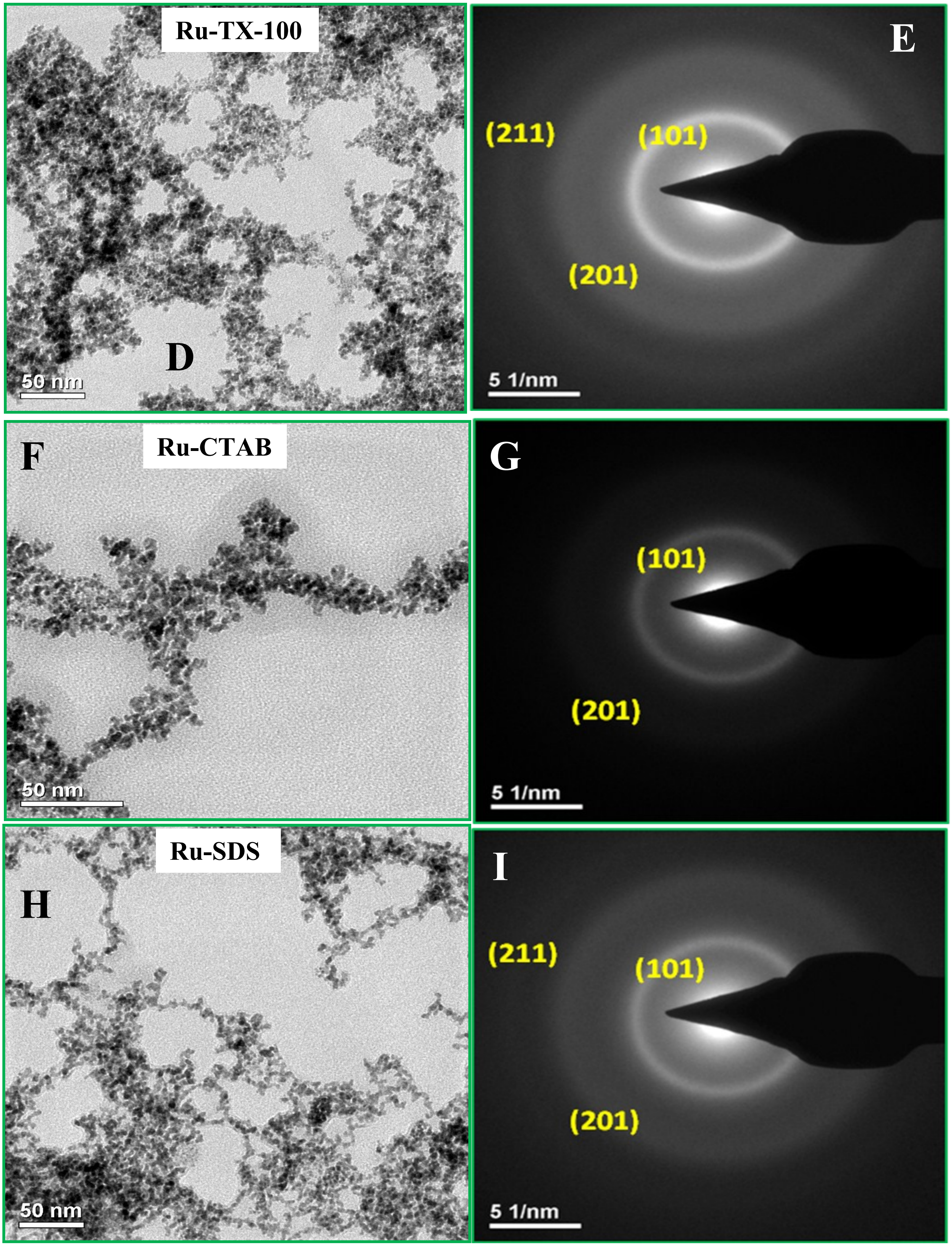
Ru-TX-100
D
50 nm
E
(211)
(101)
(201)
5 1/nm
F
Ru-CTAB
50 nm
G
(101)
(201)
5 1/nm
Ru-SDS
H
50 nm
I
(211)
(101)
(201)
5 1/nm

Figure S6, D-I

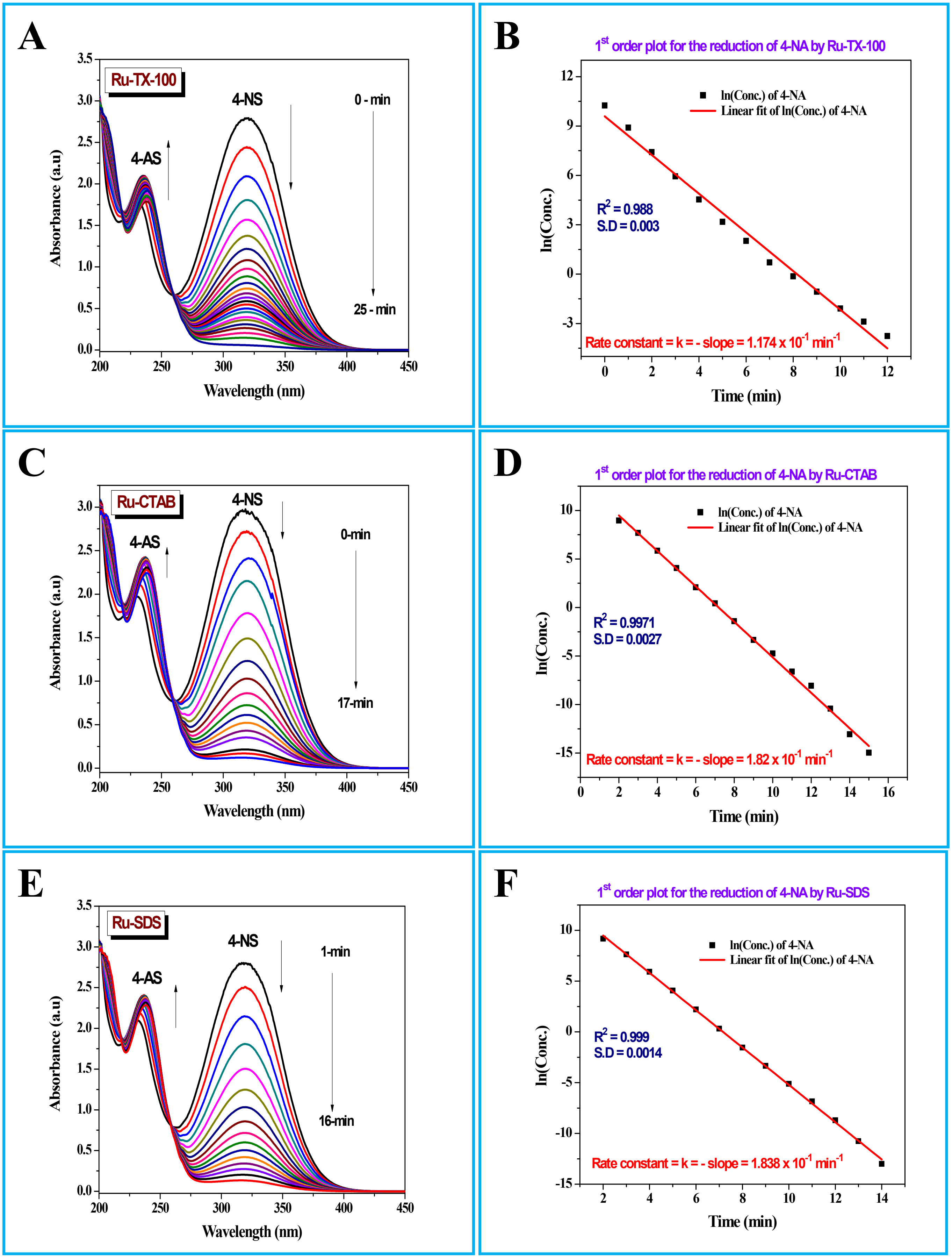
A
Ru-TX-100
4-NS
4-AS
0 - min
25 - min
Absorbance (a.u)
Wavelength (nm)
B
1st order plot for the reduction of 4-NA by Ru-TX-100
ln(Conc.) of 4-NA
Linear fit of ln(Conc.) of 4-NA
R2 = 0.988
S.D = 0.003
Rate constant = k = - slope = 1.174 x 10-1 min-1
ln(Conc.)
Time (min)
C
Ru-CTAB
4-NS
4-AS
0-min
17-min
Absorbance (a.u)
Wavelength (nm)
D
1st order plot for the reduction of 4-NA by Ru-CTAB
ln(Conc.) of 4-NA
Linear fit of ln(Conc.) of 4-NA
R2 = 0.9971
S.D = 0.0027
Rate constant = k = - slope = 1.82 x 10-1 min-1
ln(Conc.)
Time (min)
E
Ru-SDS
4-NS
4-AS
1-min
16-min
Absorbance (a.u)
Wavelength (nm)
F
1st order plot for the reduction of 4-NA by Ru-SDS
ln(Conc.) of 4-NA
Linear fit of ln(Conc.) of 4-NA
R2 = 0.999
S.D = 0.0014
Rate constant = k = - slope = 1.838 x 10-1 min-1
ln(Conc.)
Time (min)

Figure S7, A-F

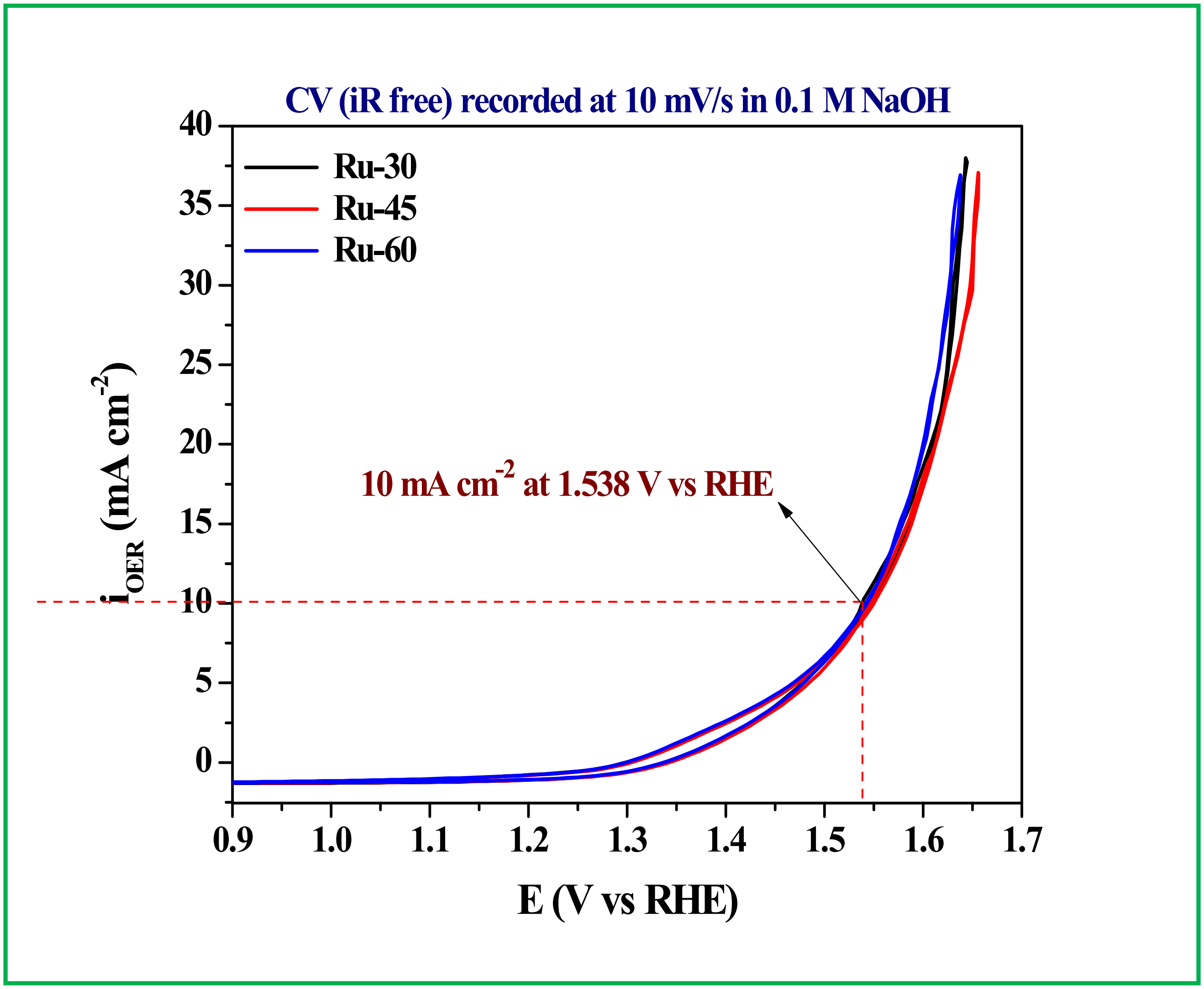
CV (iR free) recorded at 10 mV/s in 0.1 M NaOH
Ru-30
Ru-45
Ru-60
10 mA cm-2 at 1.538 V vs RHE
i_OER (mA cm-2)
E (V vs RHE)
0
5
10
15
20
25
30
35
40
0.9
1.0
1.1
1.2
1.3
1.4
1.5
1.6
1.7

Figure S8

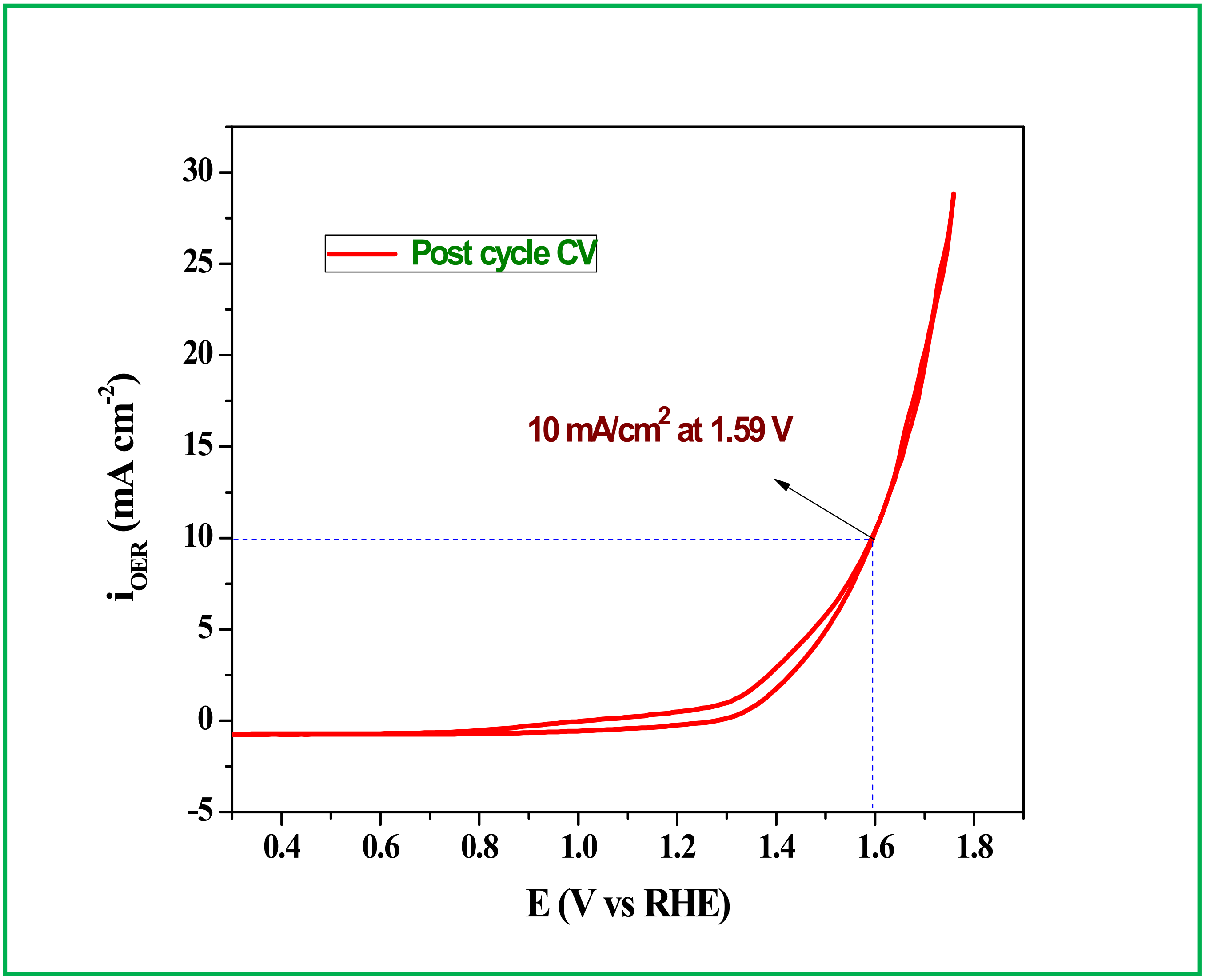

Post cycle CV
10 mA/cm2 at 1.59 V
-5
0
5
10
15
20
25
30
0.4
0.6
0.8
1.0
1.2
1.4
1.6
1.8
E (V vs RHE)
i_OER (mA cm-2)

Figure S9

S.No	Name of the nitro compound	Concentrations (M)
1	**4-Nitrophenol**	4×10^{-5}; 5×10^{-5}; 6×10^{-5}; 7×10^{-5}; 8×10^{-5}; 9×10^{-5}
2	**4-Nitroaniline**	1×10^{-5}; 2×10^{-5}; 3×10^{-5}; 4×10^{-5}; 5×10^{-5}; 6×10^{-5}; 7×10^{-5}; 8×10^{-5}; 9×10^{-5}
3	**4-Nitrostyrene**	4×10^{-7}; 5×10^{-7}; 6×10^{-7}; 7×10^{-7}; 8×10^{-7}; 9×10^{-7}; 1×10^{-6}
4	**2-Nitrophenol**	4×10^{-4}; 5×10^{-4}; 6×10^{-4}; 7×10^{-4}; 8×10^{-4}; 9×10^{-4}; 1×10^{-3}
5	**2-Nitroaniline**	1×10^{-4}; 2×10^{-4}; 3×10^{-4}; 4×10^{-4}; 5×10^{-4}; 6×10^{-4}
6	**2-Bromo-6-Nitrotoluene**	5×10^{-4}; 6×10^{-4}; 7×10^{-4}; 8×10^{-4}; 9×10^{-4}; 1×10^{-3}; 1.1×10^{-3}

Table S1

S.No	Volume, Conc. & Name of the Ar-NO_2	Volume of Ru-30/45/60 (0.01 M) (µL)	Volume of $NaBH_4$ (0.1 M) (µL)	Final Volume (mL)
1	0.5 mL of 10^{-3} M 4-NA	20	100	5
2	0.5 mL of 10^{-3} M 4-NP	20	100	5
3	0.5 mL of 10^{-6} M 4-NS	20	100	7
4	0.5 mL of 10^{-3} M 2-NP	20	100	5
5	0.5 mL of 10^{-3} M 2-NA	20	100	5
6	0.5 mL of 10^{-3} M 2-B-6-NT	20 (with Ru-60 alone)	100	5
7	0.5 mL of 10^{-3} M NB	20	100	5

Table S2

Nature of Ru catalyst	Reactant	Product	Reductant & Conditions	Time	k (min^{-1})	Yield (%)	Ref.
Ru@MMT clay	$X-C_6H_4-NO_2$ (X = H, Cl, CH_3, F, Br, CN, NH_2, OH, $-CH_3$)	$X-C_6H_4-NH_2$ (X = H, Cl, CH_3, F, Br, CN, NH_2, OH, $-OCH_3$)	iPrOH, NaOH, Reflux	4-12 h	#NA	56-97	31
Ru@CNTs & CNFs	$Cl-C_6H_4-NO_2$	$Cl-C_6H_4-NH_2$	H_2 35 bar, 60 °C	2 h	31.66*	92-94	59
Ru@Al_2O_3 Fluka	$Cl-C_6H_4-NO_2$	$Cl-C_6H_4-NH_2$	H_2 35 bar, 60 °C	2 h	#NA	100	59
Ru@Al_2O_3 Aldrich	$Cl-C_6H_4-NO_2$	$Cl-C_6H_4-NH_2$	H_2 35 bar, 60 °C	2 h	#NA	98	59
Ru@Al_2O_3 Engelhard	$Cl-C_6H_4-NO_2$	$Cl-C_6H_4-NH_2$	H_2 35 bar, 60 °C	2 h	#NA	96	59
Dendrimer encapsulated Ru	$HO-C_6H_4-NO_2$	$HO-C_6H_4-NH_2$	$NaBH_4$	20 min	3.8×10^{-1}	#NA	73
Ru@reduced graphene	$X-C_6H_4-NO_2$ (X = H, Cl, CH_3, F, Br, OH, $-OCH_3$)	$X-C_6H_4-NH_2$ (X = H, Cl, CH_3, F, Br, OH, $-OCH_3$)	$H_2$3.0 MPa 60 °C 2h	2-3 h	7*	21.6 - 99.2	56
Ru NPs	$Cl-C_6H_4-NO_2$	$Cl-C_6H_4-NH_2$	EtOH, NaOH,	48 h	#NA	16-99	40
$RuCl_2(PPh_3)_2$	$R-NO_2$	$R-NH_2$	KOH, Zn(3.3 eq), H2O 40° C	16 h	#NA	28-97	72
Unprotected Ru^0 nanochains	$X-C_6H_4-NO_2$ (X = OH, NH_2, $-CH=CH_2$, OCH_3)	$X-C_6H_4-NH_2$ (X = OH, NH_2, $-CH=CH_2$, OCH_3)	$NaBH_4$	7-16 min	2.2-4.8×10^{-1}	100	PW*

Note: #NA - 'Not available' indicates that there was no data available for k in the cited reports. *k values were calculated from the given TOF of the catalysts indirectly.

Table S3

**Ref.**	**Catalyst**	**k_{1st} (min^{-1})**	**Ref.**	**Catalyst**	**k_{1st} (min^{-1})**
3	**Au@Silica**[3]	6.83×10^{-1}	4	Pt NWs[4]	9.6×10^{-5}
5	**Au NPS**[5]	$2.7 – 5.5\times10^{-1}$	6	Au@Cellulose[6]	3.06×10^{-1}
7	**Au NPs**[7]	1.8×10^{-1}	8	Pd in microgels[8]	$0.9\text{-}2.6\times10^{-1}$
7	**Pt NPs**[7]	1.2×10^{-1}	9	Pt NPs[9]	5.7×10^{-1}
11	**Au@Ferrite**[11]	2.7×10^{-1}	10	Pd@Oxygeneous carbon[10]	5.29×10^{-1}
13	**Ag@collogen fibre**[13]	1.416×10^{-1}	12	Ni@carbon[12]	$0.906–3.75 \times10^{-1}$
14	**Au@Polymer**[14]	1.14	15	Spongy Au[15]	1.26×10^{-1}
16	**Au-Ag core-shell**[16]	2.98×10^{-1}	PW*	**Unprotected Ru^0nanochains (*Present work)**	**2.8×10^{-1}**

Table S4

References as used in the SI.

(1) Karthik, E. P.; Raja, A. K.; Kumar, S. S.; Phani, K. L. N.; Liu, Y.; Guo, S.-X.; Zhang, J.; Bond, A. M. Electroless Deposition of Iridium Oxide Nanoparticles Promoted by Condensation of $[Ir(OH)_6]^{2-}$ on Anodized Au Surface: Application to Electrocatalysis of the Oxygen Evolution Reaction. *RSC Adv.***2015**.*5*, 5196.

(2) Mccrory, C. C. L.; Jung, S.; Peters, J. C.; Jaramillo, T. F. Benchmarking Heterogeneous Electrocatalysts for the Oxygen Evolution Reaction. *J. Am. Chem. Soc.***2013**, *135*, 16977.

(3) Zhang, Z.; Shao, C.; Zou, P.; Zhang, P.; Zhang, M.; Mu, J.; Guo, Z.; Li, X.; Wang, C.; Liu, Y. In Situ Assembly of Well-Dispersed Gold Nanoparticles on Electrospun Silica Nanotubes for Catalytic Reduction of 4-Nitrophenol. *Chem. Commun.* **2011**, *47*, 3906.

(4) Qin, G. W.; Pei, W.; Ma, X.; Xu, X.; Ren, Y.; Sun, W.; Zuo, L. Enhanced Catalytic Activity of Pt Nanomaterials: From Monodisperse Nanoparticles to Self-Organized Nanoparticle-Linked Nanowires. *J. Phys. Chem. C***2010**, *114*, 6909.

(5) Gangula, A.; Podila, R.; M, R.; Karanam, L.; Janardhana, C.; Rao, A. M. Catalytic Reduction of 4-Nitrophenol Using Biogenic Gold and Silver Nanoparticles Derived from Breynia Rhamnoides. *Langmuir***2011**, *27*, 15268.

(6) Lam, E.; Hrapovic, S.; Majid, E.; Chong, J. H.; Luong, J. H. T. Catalysis Using Gold Nanoparticles Decorated on Nanocrystalline Cellulose. *Nanoscale***2012**, *4*, 997.

(7) Wunder, S.; Polzer, F.; Lu, Y.; Mei, Y.; Ballauff, M. Kinetic Analysis of Catalytic Reduction of 4-Nitrophenol by Metallic Nanoparticles Immobilized in Spherical Polyelectrolyte Brushes. *J. Phys. Chem. C***2010**, *114*, 8814.

(8) Mei, Y.; Lu, Y.; Polzer, F.; Ballauff, M.; Drechsler, M. Catalytic Activity of Palladium Nanoparticles Encapsulated in Spherical Polyelectrolyte Brushes and Core - Shell Microgels. *Chem. Mater.***2007**, *19*, 1062.

(9) Mei, Y.; Sharma, G.; Lu, Y.; Ballauff, M.; Drechsler, M.; Irrgang, T.; Kempe, R. High Catalytic Activity of Platinum Nanoparticles Immobilized on Spherical Polyelectrolyte Brushes. *Langmuir***2005**, *21*, 12229.

(10) Fang, Y.; Wang, E. Simple and Direct Synthesis of Oxygenous Carbon Supported Palladium Nanoparticles with High Catalytic Activity. *Nanoscale***2013**, *5*, 1843.

(11) Ai, L.; Yue, H.; Jiang, J. Environmentally Friendly Light-Driven Synthesis of Ag Nanoparticles in Situ Grown on Magnetically Separable Biohydrogels as Highly Active and Recyclable Catalysts for 4-Nitrophenol Reduction. *J. Mater. Chem.***012**, *22*, 23447.

(12) Yang, Y.; Ren, Y.; Sun, C.; Hao, S. Facile Route Fabrication of Nickel Based Mesoporous Carbons with High Catalytic Performance towards 4-Nitrophenol Reduction. *Green Chem.***2014**, *16*, 2273.

(13) Wu, H.; Huang, X.; Gao, M.; Liao, X.; Shi, B. Polyphenol-Grafted Collagen Fiber as Reductant and Stabilizer for One-Step Synthesis of Size-Controlled Gold Nanoparticles and Their Catalytic Application to 4-Nitrophenol Reduction. *Green Chem.***2011**, *13*, 651.

(14) Zhang, J.; Han, D.; Zhang, H.; Chaker, M.; Zhao, Y.; Ma, D. In Situ Recyclable Gold Nanoparticles Using CO_2-Switchable Polymers for Catalytic Reduction of 4-Nitrophenol. *Chem. Commun.***2012**, *48*, 11510.

(15) Rashid, H.; Bhattacharjee, R. R.; Kotal, A.; Mandal, T. K. Synthesis of Spongy Gold Nanocrystals with Pronounced Catalytic Activities Synthesis of Spongy Gold Nanocrystals with Pronounced Catalytic Activities. *Society***2006**, *22*, 7141.

(16) Jiang, H.; Akita, T.; Ishida, T. Synergistic Catalysis of Au@Ag Core−shell Nanoparticles Stabilized on Metal−organic Framework. *J. Am. Chem. Soc.***2011**, *133*,1304.
